# Multidimensional behavioral signatures of *Culex quinquefasciatus* (Diptera: Culicidae) in response to botanical volatiles reveal divergent patterns of mosquito displacement and behavioral disturbance

**DOI:** 10.1093/jme/tjag168

**Published:** 2026-11-06

**Authors:** Karthikeyan Chandrasegaran, Elspeth Cannell, David A Popko, Alec C Gerry

**Affiliations:** Department of Entomology, University of California at Riverside, Riverside, CA, USA; Department of Entomology, University of California at Riverside, Riverside, CA, USA; Department of Entomology, University of California at Riverside, Riverside, CA, USA; Department of Entomology, University of California at Riverside, Riverside, CA, USA

**Keywords:** spatial repellency, excito-repellency, essential oil, underground storm drain system, volatile organic compound

## Abstract

Underground storm drain systems (USDS) are productive but under-targeted habitats for *Culex quinquefasciatus*, where high organic loads, insecticide resistance, and odor-rich conditions complicate mosquito control. Spatial repellents offer a promising alternative, yet “repellency” encompasses multiple behavioral modes that remain poorly resolved for botanical volatiles. Here, we evaluated 4 individual plant-derived compounds, 2 whole essential oils, selected vapor-phase mixtures, and USDS water odors using a modified excito-repellency chamber to quantify escape, flying, and walking behaviors in *Cx. quinquefasciatus*. Botanical oils produced distinct, time-dependent behavioral profiles. Eugenol, citronellol, and geraniol primarily elicited escape-dominant responses consistent with spatial repellency, whereas flight activity under peppermint oil varied with active tube exposure level and time, and was accompanied by comparatively weaker escape responses. Mixture responses generally resembled the behavioral profile of the more strongly represented component rather than showing a clearly intermediate pattern. Joint escape–flight trajectories showed that treatments also differed in the temporal organization of these responses, ranging from rapid, sustained displacement to early within-chamber activation followed by delayed escape. Sewer water odors attenuated the magnitude of escape and flight responses but preserved the relative ordering of treatment-specific behavioral profiles. These findings demonstrate that botanical volatiles can produce distinct behavioral response profiles that remain detectable in a chemically complex odor environment. By repeatedly quantifying escape, flight, and walking activity within a single experimental system, this study provides a comparative behavioral framework to identify volatile treatments that warrant further evaluation to alter adult mosquito retention in odor-rich urban infrastructures, such as underground storm drains.

## Graphical Abstract Graphical abstract

**Graphical Abstract:**
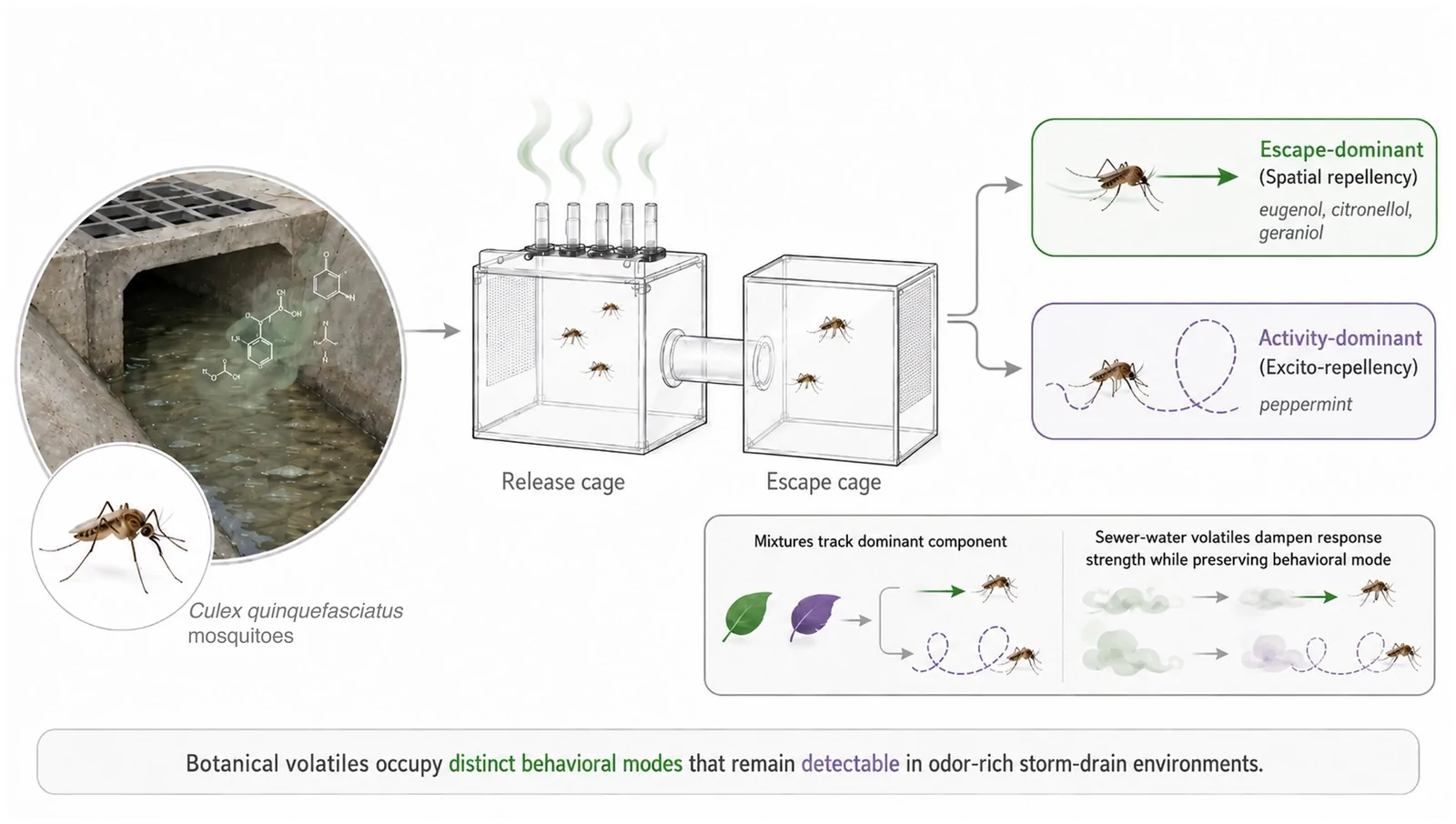

## Introduction

Mosquito-borne diseases remain a major global health burden, causing substantial mortality, long-term morbidity, and socioeconomic disruption across tropical, subtropical, and increasingly urban regions (Achee et al. 2012). Rapid urbanization has intensified this challenge by generating built environments that inadvertently support mosquito proliferation. Among these, Underground Storm Drain Systems (USDS) have emerged as highly productive yet under-recognized habitats (Rydzanicz et al. 2016, Wang et al. 2021). These structures maintain stable darkness, high humidity, minimal airflow, and persistent pools of organic-rich water that strongly favor the development of *Culex quinquefasciatus*, a primary vector of West Nile virus and lymphatic filariasis (Strickman and Lang 1986, Reisen et al. 1990, Suman et al. 2022). USDS therefore functions not only as larval habitat but also as a protected refugium for resting adults, linking engineered urban infrastructure to an elevated local risk of pathogen transmission.

Controlling mosquitoes within the USDS is operationally difficult. Hydrological flushing reduces the persistence of larvicides, and high organic loads diminish the performance of microbial agents such as *Bti* (Estrada and Mulla 1986, Des Lauriers et al. 2006). At the same time, *Cx. quinquefasciatus* populations in many regions exhibit substantial resistance to organophosphates, carbamates, and pyrethroids (Bisset et al. 1990, Peiris and Hemingway 1993, Low et al. 2013). These limitations have intensified interest in complementary tools that act through behavioral disruption rather than direct toxicity. Spatial repellents (SRs), which alter mosquito host-seeking, resting, or entry behaviors through airborne volatiles applied to USDS or other engineered structures in urban environments, represent 1 promising class of such interventions.

Most applications of botanical repellents have focused on personal protection or prevention of mosquito contact with humans. In contrast, enclosed infrastructures such as USDS may provide an opportunity for area-based behavioral intervention. Adult *Cx. quinquefasciatus* use these structures as protected resting and refuge sites, while females may also enter or remain near standing water associated with immature development. Volatile treatments that promote departure from these structures, discourage prolonged rest, or otherwise disrupt adult retention could complement larval control and conventional adulticide applications, particularly where access is limited and organic contamination reduces insecticide performance. However, the behavioral properties required for such an application remain poorly defined, and responses measured under conventional personal-repellent assays may not translate directly to enclosed, odor-rich environments.

Although SRs are often treated as a single functional category, volatile treatments may produce several distinguishable behavioral outcomes. These include spatial displacement, expressed as movement away from or departure from a treated space, and behavioral activation or disturbance, expressed as increased locomotor activity without demonstrated directional movement away from the stimulus (Noosidum et al. 2008, Boonyuan et al. 2014, Nararak et al. 2019). In the present study, departure from the treated release cage was interpreted as spatial displacement, whereas flight and walking activity among mosquitoes that remained within the cage were interpreted as behavioral activation or disturbance. Although such activity is commonly discussed within excito-repellency frameworks, increased activity alone was not considered direct evidence of repellency because it did not demonstrate directional movement away from the volatile source (Dethier et al. 1960). Distinct modes of action may differentially influence mosquito microhabitat use, occupancy of sheltered structures, or deterrence from resting sites (Norris and Coats 2017, Achee and Grieco 2018, 2022, Achee et al. 2023). Yet, for many candidate repellents, including botanical essential oils, the behavioral dimensions that underpin “repellency” remain incompletely characterized, limiting the ability to predict performance across environments.

Plant-derived volatile compounds and whole essential oils have received considerable attention as potential alternatives to synthetic mosquito repellents. Individual constituents such as cinnamaldehyde, citronellol, eugenol, and geraniol, as well as complex essential oils such as geranium and peppermint oils, can alter mosquito host-location, landing, probing, and resting behavior (Nerio et al. 2010, Senthil-Nathan 2020, Popescu et al. 2024). These botanical treatments have demonstrated repellent or activity-modifying effects in laboratory and semi-field assays (Ansari et al. 2000, Peach et al. 2019, Sutthanont et al. 2022). However, individual compounds and whole essential oils differ in composition, volatility, sensory effects, and behavioral activity. Mixtures of botanical volatiles may also produce response profiles that are not readily predicted from those of the individual components. These differences suggest that botanical treatments may produce distinct behavioral outcomes, but a consistent framework for comparing these responses remains limited.

USDS environments introduce an additional, largely unexplored complexity to the application of mosquito-repellent materials due to a dense background of volatile organic compounds (VOCs) generated by microbial decomposition and stagnant wastewater. Background odors can mask, compete with, or reshape the sensory coding of repellent volatiles (Afify et al. 2019, Afify and Potter 2020), potentially altering the expression or detectability of spatial repellence or excito-repellency behaviors. Despite the relevance of odor complexity to real-world mosquito behavior, little is known about how environmental VOCs influence essential oil–based repellents, particularly in enclosed urban structures such as storm drains.

This study addresses these gaps by evaluating botanical volatiles and their mixtures as candidate behavioral-modifying treatments for *Cx. quinquefasciatus* under controlled conditions relevant to USDS environments. Although the behavioral responses of mosquitoes following exposure to plant-derived compounds have been investigated previously, the present study applies a modified excito-repellency chamber to resolve multiple components of mosquito response within a single experimental framework. We quantified cumulative departure from the treated chamber, flying and walking activity among mosquitoes that remained within the chamber, and vertical redistribution through repeated observations over time. This framework was used to compare individual botanical compounds and whole essential oils, evaluate how active-tube exposure level and vapor-phase mixture composition influenced behavioral profiles, and determine whether odors associated with USDS water modified responses to selected treatments. By distinguishing displacement from within-chamber behavioral activation, this study provides an integrated comparative approach to characterize the multidimensional effects of botanical volatiles and identify treatments that warrant further evaluation to reduce adult mosquito retention or use of odor-rich urban infrastructures such as USDS.

## Materials and Methods

### Mosquito Rearing

*Cx. quinquefasciatus* mosquitoes used in this study were derived from a laboratory colony originally established from field collections in the Inland Empire region of Southern California for a previous investigation of urban storm drain–associated populations (Popko et al. 2018). This regionally derived strain is ecologically relevant because *Cx. quinquefasciatus* in southern California commonly exploit underground storm drain systems and other organically enriched urban aquatic habitats characterized by chemically complex odor environments. Using a colony founded from these populations enabled evaluation of behavioral responses in a genetic background representative of mosquitoes that naturally encounter sewer- and drainage-associated volatile cues. The colony was maintained under controlled laboratory conditions, including a temperature of 26 ± 1 °C, relative humidity of 70% ± 10%, and a 12:12 light-dark cycle. Adult females aged 5 to 9 days that were nulliparous and non-blood-fed were selected as a manageable, physiologically standardized, and operationally important cohort. Restricting assays to females of a defined age and reproductive state reduced variation associated with age, parity, blood feeding, and gonotrophic condition, thereby allowing treatment-specific behavioral responses to be compared under consistent laboratory conditions. Approximately 30 females were assigned to each assay group on the day preceding behavioral testing. Mosquitoes were cold-immobilized on ice (∼4 °C) for 15 to 20 min to facilitate transfer into white paper holding cups (WebstaurantStore, White Poly-Coated Paper Food Cup, 16 oz.) with a mesh top. The transferred mosquitoes were subsequently maintained under colony conditions for approximately 12 h, during which sucrose was withheld and water was provided. This 12 h recovery interval was incorporated to minimize any residual effects of cold immobilization on subsequent behavioral responses. On the day of testing, mosquitoes were observed to be active and were not chilled or otherwise immobilized.

### Repellent Compounds

The study evaluated 4 individual plant-derived compounds, cinnamaldehyde, citronellol, eugenol, and geraniol, and 2 whole essential oils, geranium oil and peppermint oil. *N*,*N*-diethyl-meta-toluamide (DEET) was included as a synthetic positive control, and mineral oil was used as the negative control. Treatments were selected based on previous evidence of behavioral activity against mosquitoes (Hao et al. 2014, Nerio et al. 2010, Deletre et al. 2015). All compounds, essential oils, DEET, and mineral oil were used as supplied by the manufacturer without further dilution. Supplier and product information are provided in Table S1. Eugenol and peppermint oil were also evaluated at fixed active-tube ratios to characterize mixture-specific behavioral responses. These assays were not designed as formal tests of additive, synergistic, or antagonistic interactions.

### Modified Excito-Repellency Chamber Assays

Behavioral responses to volatile treatments were assessed using a modified excito-repellency chamber (Fig. 1) based on Roberts et al. (1997). The assay chamber included 2 compartments: a release cage, where mosquitoes were initially introduced and exposed to volatile treatments, and an escape cage connected by a 6-inch-diameter PVC pipe that served as the escape port. The top of the release cage contained 5 slots to hold 15 ml plastic tubes (Falcon^TM^ 15 ml Conical Centrifuge Tubes, Corning), which were perforated along the upper half of each tube to allow release of volatiles from the compounds within. Each of the 5 tube positions contained either a dummy tube or an active volatile-release tube containing a cotton wick saturated with approximately 6 ml of the test material.

**Fig. 1.**
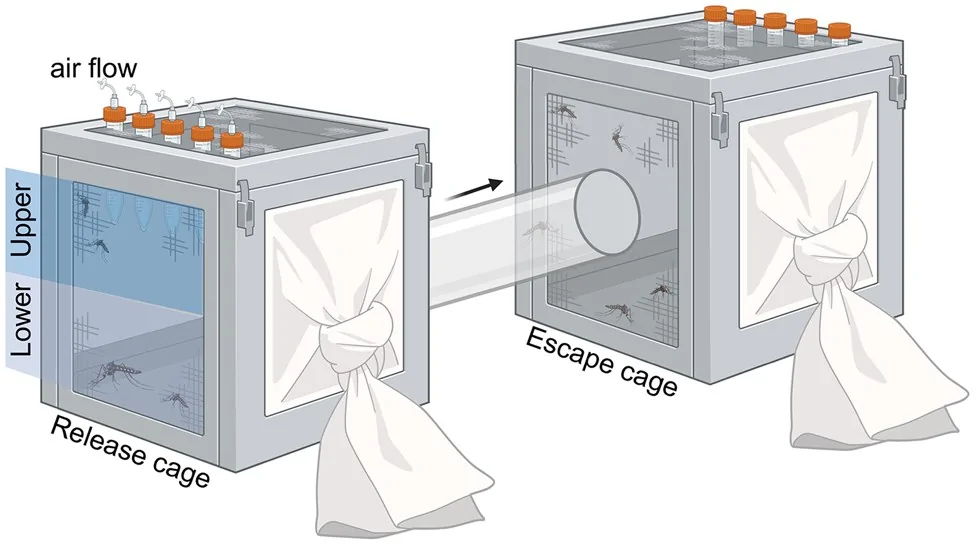
Modified excito-repellency chamber for quantifying spatial displacement and within-cage behavioral activity. Schematic representation of the dual-compartment chamber used to evaluate mosquito responses to volatile treatments, showing the treated release cage, connecting tube, escape cage, and direction of airflow. The chamber was used to distinguish departure from the treated release cage from flying, walking, and vertical redistribution among mosquitoes that remained within it. Mosquitoes in the connecting tube and escape cage were recorded as occupying separate locations but were pooled for the primary escape endpoint because both represented departure from the treated release cage. Connecting-tube occupancy was transient, and no mosquitoes were observed returning to the release cage after entering the tube. Flying and walking within the release cage were interpreted as behavioral activation or disturbance rather than direct evidence of spatial repellency. The schematic is not drawn to scale.

Outside air was supplied through a vinyl-tubing circuit connected to each volatile-release tube. Air velocity was measured at the outlet of each tube using a Kestrel 5500 Weather Meter (Kestrel Instruments, Boothwyn, PA, USA). Before commencing experiments each day, measurements were obtained in-line (via vinyl tubing) before air entered each of the 5 tube outlets, and the mean air velocity entering each volatile release tube was 0.75 ± 0.094 m s^−^^1^ (mean ± SD). This configuration was used to promote consistent delivery of volatiles from the release tubes into the release cage. The release cage was covered with plastic wrap to contain volatiles, whereas the escape cage remained uncovered. Ambient laboratory lighting remained uniform across the assay apparatus throughout all trials.

Each holding cup was placed inside the release cage and opened, allowing mosquitoes to enter the assay chamber voluntarily. After all mosquitoes had exited the cup, the cup was removed, and mosquitoes were allowed to acclimate to the release cage for 5 min before introduction of the volatile-release tubes. This 5-min period was intended to allow acclimation to the assay arena and was not used for recovery from cold immobilization. Identical handling, holding, transfer, and acclimation procedures were used for all treatment and control trials.

For each assay, approximately 30 female mosquitoes were introduced into the release cage containing only dummy tubes. Treatment exposure was initiated by replacing 1, 3, or 5 dummy tubes with active volatile-release tubes containing the test material; the remaining dummy tubes were left in all unused positions. Nominal exposure level was operationally defined by the number of active treatment tubes deployed among the 5 available tube positions. Because airborne chemical concentrations and compound-specific emission rates were not quantified, these conditions are referred to as 1-, 3-, and 5-active-tube exposure levels rather than as chemical concentrations or doses. The initial screening assay was conducted using 5 active treatment tubes.

A single assay trial, comprising 1 cohort of approximately 30 females, constituted the independent experimental unit. Individual mosquitoes and repeated observations of the same cohort were not treated as independent replicates. In the initial 5-active-tube screening, 4 independent trials were conducted for geraniol, 6 for peppermint oil, and 5 for each of the remaining treatments. Five trials were conducted for each treatment-by-exposure-level combination, except the 5-active-tube peppermint oil condition, which included 6 trials. Five independent trials were also conducted for each volatile mixture treatment and for each volatile treatment evaluated in the presence of sewer-water volatiles.

Mosquito behavior and location were observed at 5, 10, 20, 30, and 40 min after introduction of the treatment. At each observation time, mosquitoes were assigned to 1 of 4 mutually exclusive spatial locations: the upper release cage, lower release cage, connecting tube, or escape cage (Fig. 1). Occupancy of the upper and lower sections of the release cage was retained as a separate measure of vertical redistribution and was not included in the pooled escape endpoint. For the primary analyses, mosquitoes occupying either the connecting tube or escape cage were classified as having departed (escaped) the release cage. This pooled escape endpoint was defined a priori because entry into the connecting tube indicated displacement from the treated chamber and movement into the designated escape pathway. Tube occupancy was transient, generally lasting from several seconds to a few minutes, and mosquitoes that entered the tube were not observed returning to the release cage; instead, they subsequently progressed into the escape cage.

The chamber was used to quantify 2 operationally distinct categories of behavioral response. Movement from the treated release cage into the connecting tube or escape cage was interpreted as spatial displacement because it represented departure from the treated compartment. In contrast, flying and walking recorded among mosquitoes that remained within the release cage were interpreted as within-chamber behavioral activation or disturbance. Because these activity responses did not necessarily involve movement away from the volatile source, they were not considered direct evidence of spatial repellency. Mosquitoes occupying the connecting tube or escape cage were classified under the escape endpoint and were not simultaneously scored as flying or walking. At the end of each assay, mosquitoes were transferred to clean holding containers and maintained under standard laboratory conditions. At 24 h post-exposure, the number of dead females was recorded, and trial-level mortality was calculated relative to the initial assay cohort. Blank mortality entries, i.e. trials lacking mortality observations, were treated as missing observations rather than as zero mortality.

To evaluate mixture-specific behavioral responses, eugenol and peppermint oil were tested in 2 fixed active-tube ratios. The 3:1 treatment consisted of 3 active tubes containing eugenol and 1 active tube containing peppermint oil, whereas the 1:3 treatment consisted of 1 active tube containing eugenol and 3 active tubes containing peppermint oil. Each mixture treatment therefore contained 4 active treatment tubes, with 1 dummy tube occupying the remaining fifth position. This configuration allowed the vapors to mix within the release cage while preventing direct liquid-phase interaction between the treatments. Escape, flying, and walking responses under each mixture treatment were compared descriptively with those observed under the corresponding individual treatments to determine whether mixture profiles were intermediate or more closely resembled 1 component. Because airborne concentrations and component-specific emission rates were not quantified, and because the study did not include individual-component treatments matched to the mixture configurations or specify an additive null model, the experiment was not designed to formally distinguish among additive, synergistic, and antagonistic interactions. Five independent trials were conducted for each mixture treatment.

### Sewer Water Volatiles and Chemical Analyses

The influence of odors associated with USDS water on treatment-induced behavioral responses was assessed using eugenol and a 3:1 eugenol:peppermint oil mixture. In the USDS-water assays, eugenol was delivered from 3 active eugenol tubes, whereas the mixture consisted of 3 active eugenol tubes and 1 active peppermint-oil tube. Five independent trials were conducted for each volatile treatment under the presence of USDS-water condition. Water samples were collected and combined from 3 different USDS chambers located in a gated residential community in the city of Palm Desert, southern California. Following collection from the field, water samples were stored in airtight amber glass containers at −20 °C to preserve their chemical composition. Before each assay, 100 ml of USDS water was thawed in the sealed container at room temperature and placed in the release cage 30 min prior to the assay to allow VOCs to equilibrate within the headspace before adding active volatile release tubes containing the selected botanical volatile treatments. Escape, flight activity, and walking responses obtained in the presence of USDS water were compared with responses from the same volatile treatments in assays conducted without USDS water.

To characterize the chemical properties of pooled USDS water, measurements of pH, electrical conductivity, total organic carbon (TOC), and total nitrogen (TN) were performed. pH and electrical conductivity were measured using a benchtop multiparameter meter (Oakton PC700, Oakton Instruments, Vernon Hills, IL, USA). The instrument was calibrated prior to analysis using standard buffer solutions (pH 4.00, 7.00, and 10.00) and a 1413 µS cm^−^^1^ conductivity standard. Total organic carbon was quantified using a combustion catalytic oxidation analyzer (Shimadzu TOC-L, Shimadzu Scientific Instruments, Columbia, MD, USA). The system was calibrated with multi-point TOC standards spanning the instrument’s working range. Samples were injected into a combustion tube maintained at 720 °C containing an oxidation catalyst. Combustion-derived CO_2_ was transported via carrier gas through a dehumidifier and halogen scrubber and quantified using a non-dispersive infrared detector integrated within the instrument. Total nitrogen was measured using the same platform equipped with a nitrogen analysis module (Shimadzu TOC-L with TNM-L module, Shimadzu Scientific Instruments, Columbia, MD, USA). Nitrogen-containing compounds were thermally converted to nitrogen monoxide, which was introduced into a chemiluminescence detector for quantification following calibration with multi-point nitrogen standards.

### Data Analysis

For each trial and observation time, we calculated the proportion of the original assay cohort classified as escaped, flying, or walking. Escape was operationally defined as departure from the treated release cage and included mosquitoes occupying either the connecting tube or the escape cage. These locations were pooled because connecting-tube occupancy represented a transient, 1-way stage of movement toward the escape cage, with no observed return to the release cage. Flying and walking were scored only for mosquitoes that remained within the release cage. Thus, escape and within-cage activity represented mutually exclusive behavioral states at a given observation time.


Yadjusted,i,t=Ytreatment,i,t-Ycontrol,i,t

where *Y_treatment,i,t_* is the proportion exhibiting the behavior in treatment trial i at observation time t; *Y_control,i,t_* is the corresponding proportion in the mineral-oil control paired with that treatment trial, and *Y_adjusted,i,t_* is the resulting control adjusted response. Treatment and control trials were paired within the same experimental block and assay date. Positive values indicated a greater response under the volatile treatment, negative values indicated a lower response, and zero indicated no difference in response from the mineral oil control. These control-adjusted responses were used in all subsequent statistical analyses. Because the same trial was observed repeatedly over time, escape, flying, and walking responses were analyzed separately using linear mixed-effects models fitted with the *lmer* function in the *lme4* package. Treatment, observation time, and their interaction were included as fixed effects, and trial replicate was included as a random intercept to account for repeated measurements within trials. The same modeling framework was used to compare behavioral responses to eugenol and peppermint oil across 1-, 3-, and 5-active-tube exposure levels. Increasing the number of active tubes was expected to increase total volatile delivery, but airborne concentrations and emission rates were not quantified. Eugenol–peppermint oil mixtures were modeled separately, and their behavioral profiles were compared descriptively with those of the corresponding individual treatments. These comparisons were used to assess whether mixture responses were intermediate or more closely resembled those of a single component, and they were not treated as formal tests of additivity, synergy, or antagonism.

The effects of sewer water volatiles were assessed using mixed-effects models fitted to assays conducted in the presence of sewer water. To quantify differences associated with the presence or absence of USDS-water, replicate-level response differences were calculated separately for eugenol and the eugenol:peppermint oil 3:1 mixture as the response from assays conducted without USDS water minus the corresponding response from assays conducted in the presence of USDS water. Positive values therefore indicated a larger response without USDS water, negative values indicated a larger response in the presence of USDS water, and zero indicated no difference between conditions. These response differences were analyzed using mixed-effects models that included treatment, observation time, and their interaction as fixed effects and trial identity as a random intercept. Statistical significance was evaluated using F tests (*lmerTest* package), with *P *< 0.05.

To visualize the joint temporal expression of the 2 most responsive behavioral endpoints, control-adjusted flight activity was plotted against control-adjusted escape in a fixed 2-dimensional response space. Escape and flight activity were analyzed separately using longitudinal linear mixed-effects models with treatment and observation time as a categorical factor, and their interaction as fixed effects, and trial identity (replicate) as a random intercept. Estimated marginal means and marginal 95% confidence intervals were obtained for each treatment at 5, 10, 20, 30, and 40 min. Time-specific estimates were paired and connected chronologically to form treatment-specific trajectories; horizontal and vertical error bars represent uncertainty in flight activity and escape, respectively.

Because escaped mosquitoes were no longer scored for activity within the release cage, these trajectories represent complementary cohort-level responses rather than individual mosquito movement paths. Arrows indicate temporal order only and do not demonstrate that mosquitoes recorded flying subsequently escaped. Dashed reference lines indicate no difference from the paired mineral-oil control. The dotted diagonal indicates equal numerical magnitudes of the 2 control-adjusted responses but does not imply equivalent biological processes, equal behavioral contribution, or a physiological tradeoff. Walking was excluded from this visualization because it showed limited treatment-specific temporal structure but remained included in the behavior-specific analyses. Because individual treatments and mixtures were evaluated using different active-tube configurations, the trajectories were interpreted descriptively across assay series. Statistical inference remained based on the corresponding endpoint-specific mixed-effects models, and trajectory position or direction was not used to test behavioral classes, mixture additivity, synergy, or antagonism. All analyses were performed in R (version 4.2.2).

## Results

### Botanical Volatiles Produce Distinct Temporal Patterns of Escape and Within-Chamber Activity

Across all assays, occupancy of the connecting tube was transient. Mosquitoes entering the tube subsequently progressed into the escape cage, and no mosquitoes were observed returning to the release cage. Thus, the connecting tube did not function as a persistent alternative location during the assay. Mosquito escape responses exhibited strong, treatment-specific temporal divergence (Fig. 2A). The significant oil-by-time interaction (*F*_6,152.09_ = 2.69, *P *= 0.016) indicates that botanical volatile treatments produced unique escape trajectories rather than a shared temporal pattern. Although the proportion of mosquitoes escaping differed across oils (*F*_6,152.94_ = 4.00, *P *= 9.46 × 10^−^^4^) and changed over the course of the assay period (*F*_1,152.09_ = 15.77, *P *= 1.10 × 10^−^^4^), these patterns were not independent. The direction and magnitude of temporal change varied across oils with the escape profile often shifting as exposure progressed (Fig. 2A). Eugenol showed an early rise in escape that stabilized with few additional escapes after approximately 10 to 20 min, indicating that most net displacement relative to mineral oil occurred early in the assay and then remained steady through 40 min (*β* = 0.1057 ± 0.0453, *P *= 0.0209). In contrast, peppermint oil showed limited early escape, followed by a progressive increase in escape over time, consistent with a delayed or activity-mediated contribution to displacement rather than an immediate spatial repellent effect. Geraniol exhibited an intermediate profile, with a moderate initial escape response, lower than eugenol but higher than most other botanical volatile treatments, followed by a gradual increase in escape over time, partially resembling the trajectory observed for peppermint oil. Other botanical volatile treatments elicited little to no temporal change in escape. These divergent slopes indicate that escape, the operational measure of spatial displacement in this assay, is strongly shaped by treatment identity and its temporal progression.

**Fig. 2.**
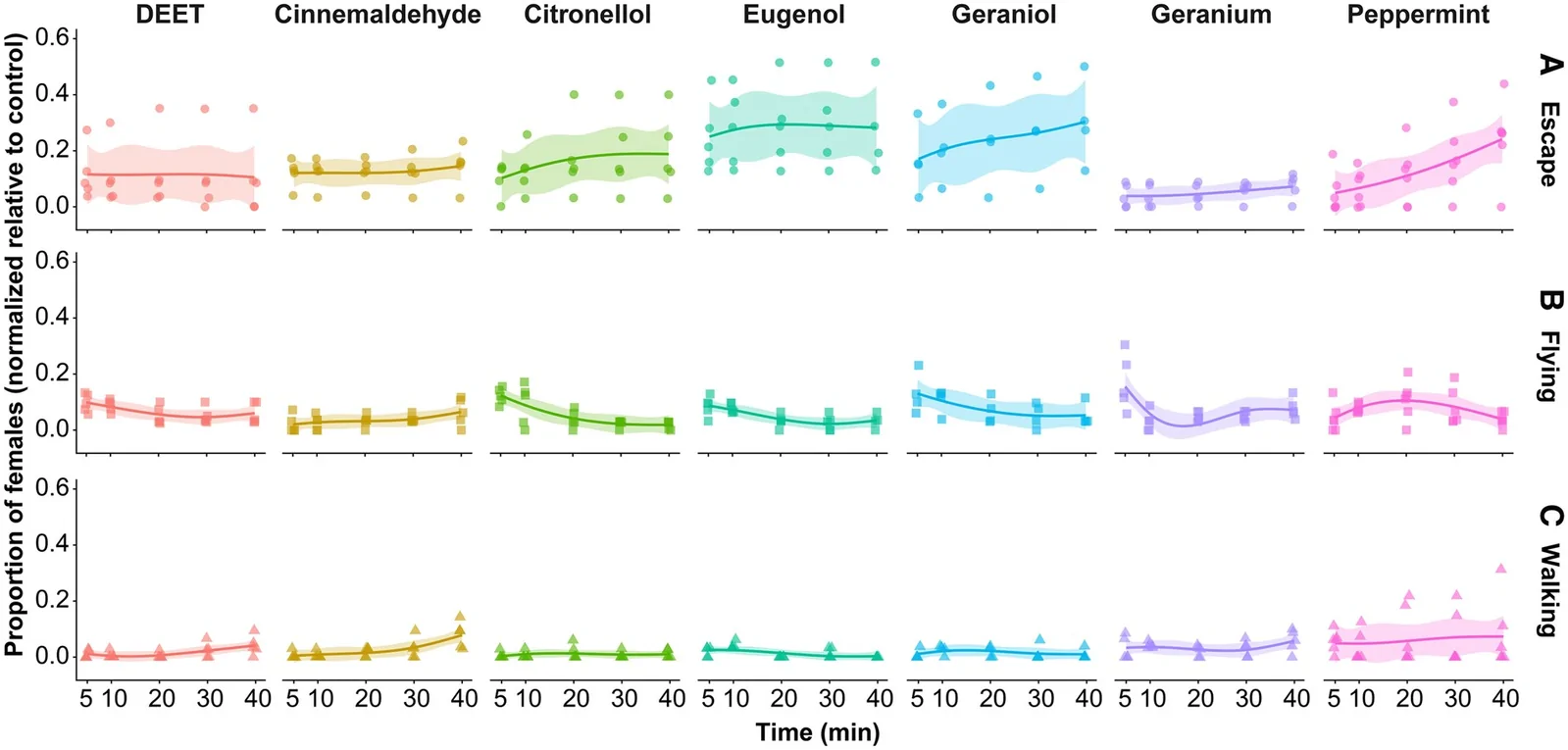
Temporal dynamics of escape, flight, and walking responses of *Culex quinquefasciatus* to botanical volatile treatments. Escape (A), flight (B), and walking (C) responses were recorded over a 40 min exposure period for 4 individual plant-derived compounds (cinnamaldehyde, citronellol, eugenol, and geraniol), 2 whole essential oils (geranium oil and peppermint oil), and the synthetic repellent DEET. Each point represents the proportion of mosquitoes exhibiting the indicated behavior within an independent trial at 5, 10, 20, 30, or 40 min, expressed as the treatment response minus the corresponding response in the paired mineral oil control at the same observation time. Values above and below zero therefore indicate responses greater than and lower than the paired control, respectively. Each assay included 23 to 40 female mosquitoes. Treatments were represented by 4 to 6 independent trials: geraniol, *n *= 4; peppermint oil, *n *= 6; and all remaining treatments, *n *= 5. Points are colored by treatment identity, and circles, squares, and triangles denote escape, flight, and walking, respectively. Solid lines show model-based temporal fits using natural splines (*df *= 3), and shaded bands represent 95% confidence intervals. All treatments were evaluated under the 5 active tube condition. Behavioral proportions were analyzed using mixed-effects models with treatment identity, time, and their interaction as fixed effects and trial identity as a random intercept to account for repeated observations within trials.

Flight activity, an indicator of within-chamber behavioral disturbance, also varied markedly by essential oil and exposure time (Fig. 2B). The robust oil-by-time interaction (*F*_6,160_ = 3.72, *P *= 0.0017) demonstrates that temporal changes in flying were highly treatment-specific. Treatment identity (*F*_6,160_ = 4.85, *P *= 1.41 × 10^−4^) and time (*F*_1,160_ = 21.75, *P *= 6.50 × 10^−6^) both shaped these responses, but in patterns that differed across oils. Among botanical volatile treatments, citronellol, geraniol, and geranium oil elicited pronounced early flight activity that declined over time, with citronellol showing the steepest negative temporal slope (*β* = −0.00411 ± 0.00099, *P *= 5.36 × 10^−5^). Although some decline in flight activity may result from mosquito escape, escape was limited beyond 10 to 20 min for most oils (except peppermint oil and geraniol), suggesting that the reduction in flight activity primarily reflects behavioral attenuation within the release cage rather than progressive loss of individuals. Other botanical oils exhibited lower flight activity with flatter or more sustained activity profiles. As a result, flight activity showed greater variation among treatments than walking activity, but less divergence than escape activity. Responses to DEET broadly aligned with the initially elevated flight activity and its subsequent decline observed for several botanical oils, providing a useful benchmark for comparing botanical volatile treatments with the synthetic spatial repellent (positive control).

Compared with escape and flight activity, walking activity exhibited the weakest response to oils and the most limited temporal structure (Fig. 2C). The oil-by-time interaction was not significant (*F*_6,152.78_ ≈ 1.0 to 1.3, *P *> 0.1), nor was the main effect of treatment identity (*F*_6,152.78_ = 1.59, *P *= 0.154). Walking activity changed only slightly over the course of the assay (*F*_1,150.30_ = 4.56, *P *= 0.034), and the few oils that showed shallow negative slopes (all *P *< 0.03) did so with very small effect sizes. Overall, walking remained the most stable and least responsive of the 3 behaviors, consistent with its narrow dynamic range across the assay. Complementary analyses of vertical occupancy within the release cage showed treatment-specific temporal patterns that broadly aligned with the flight activity responses shown in Fig. 2, suggesting that vertical redistribution primarily reflected within-chamber behavioral activation rather than a distinct avoidance response (Fig. S1).

### Active Tube Exposure Level Shapes Escape, Flight Activity, and Walking Responses to Eugenol and Peppermint Oil

Escape responses varied strongly with active tube exposure level and differed between eugenol and peppermint oil (Fig. 3A). Treatment identity (*F*_1,133.23_ = 7.38, *P *= 0.0075), time (*F*_1,132.26_ = 8.41, *P *= 0.0044), and their interaction (*F*_1,132.26_ = 4.66, *P *= 0.0327) all contributed to temporal structure, indicating that exposure level modulated escape in a treatment-specific manner. Eugenol elicited rapid, exposure-level-dependent escape, with strong displacement evident within the first 5 min at higher exposure levels and sustained thereafter. In contrast, peppermint oil exhibited minimal early escape and a gradual, time-dependent increase that became apparent primarily at the 5-tube treatment. Although escape increased over time for peppermint oil under the 5-tube treatment, this response remained significantly weaker than the corresponding exposure level-dependent increase observed for eugenol (peppermint oil × five-tube interaction: −0.2196 ± 0.0580, *P *= 0.00023). Across most observation times, escape under peppermint oil was lower than or comparable to eugenol at corresponding active-tube exposure levels, with responses appearing to converge by 40 min. Together, these results demonstrate that escape is strongly exposure level-sensitive but governed by treatment-specific temporal dynamics.

**Fig. 3.**
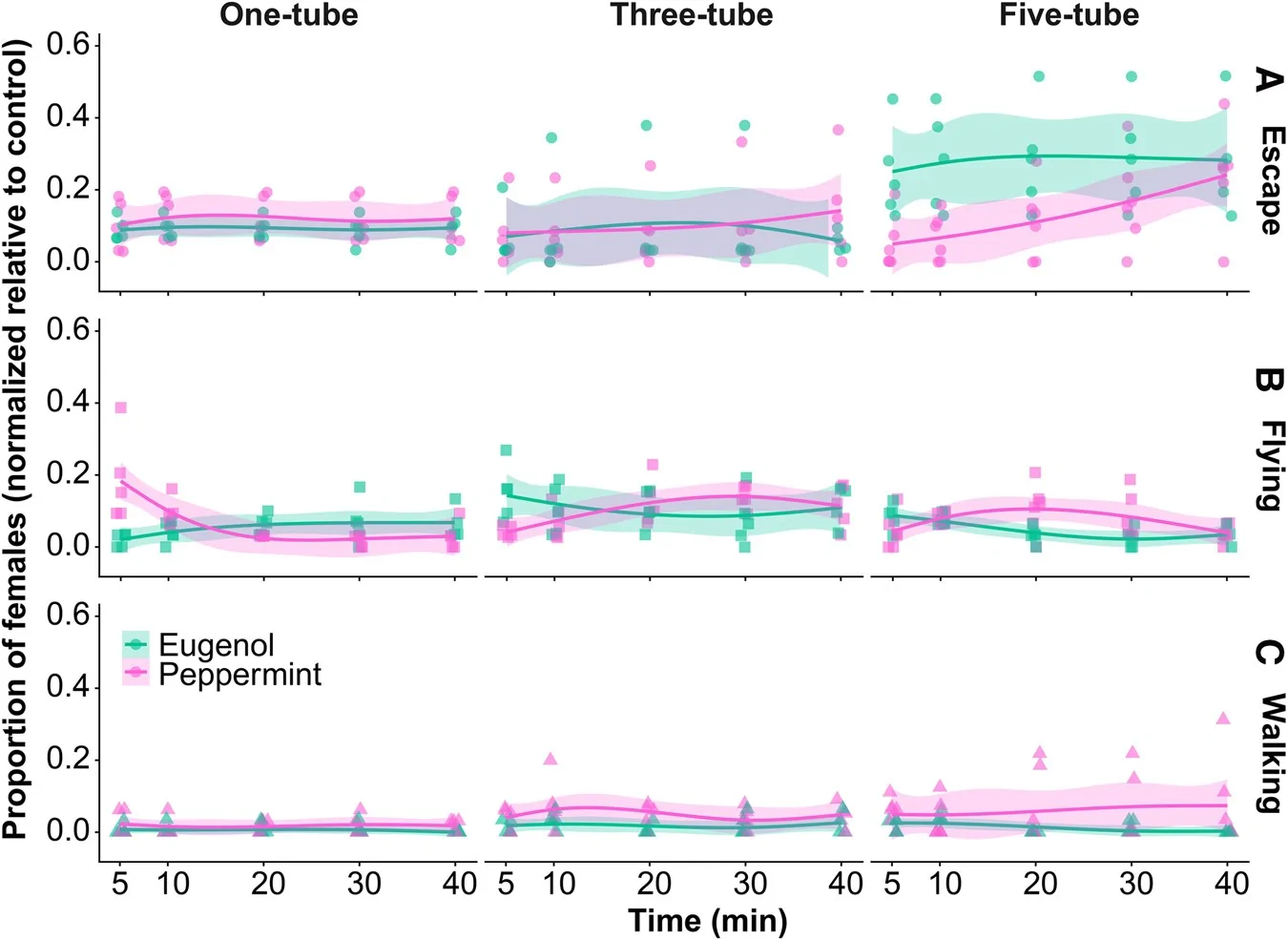
Temporal dynamics of escape, flight activity, and walking across active-tube exposure levels of eugenol and peppermint oil. Escape (A), flight (B), and walking (C) responses of *Culex quinquefasciatus* were recorded over a 40-min assay under 1-, 3-, and 5-active-tube conditions of eugenol and peppermint oil. Each point represents the proportion of mosquitoes exhibiting the indicated behavior within an independent trial at 5, 10, 20, 30, or 40 min, expressed relative to the corresponding paired mineral oil control at the same observation time. Each assay included 26 to 38 female mosquitoes. Five independent trials were conducted for each treatment and active-tube combination, except the 5-active-tube peppermint oil condition, for which 6 trials were conducted. Point shapes distinguish the 3 behavioral responses: circles (escape), squares (flight), and triangles (walking), while colors distinguish eugenol (green) and peppermint oil (magenta) treatments. Solid lines show model-based temporal fits using natural splines (*df *= 3), and shaded bands represent 95% confidence intervals. Under the 5-active-tube condition, the escape response to peppermint oil was significantly weaker than that to eugenol, as indicated by the significant treatment × 5-active-tube interaction (estimate = −0.2196 ± 0.0580, *P *= 0.00023). Behavioral responses were analyzed using mixed-effects models with treatment identity, active-tube exposure level, time, and their interactions as fixed effects and trial identity as a random intercept to account for repeated observations within trials.

Flying behavior exhibited strong exposure level-dependent dynamics that differed fundamentally from escape (Fig. 3B). While active tube exposure level influenced overall flying activity, the dominant feature was a significant 3-way interaction among treatment identity, time, and active tube exposure level (F_2,135.24_ = 16.40, *P *= 4.19 × 10^−7^), indicating that temporal trajectories in flying depended jointly on treatment identity and active tube exposure level. This pattern was driven primarily by peppermint oil, which showed a pronounced negative temporal slope in the 1-tube treatment (β = −0.00523 ± 0.00114, *P *= 1.00 × 10^−5^) but positive temporal slopes at the 3 and 5 active tube exposure levels (3-tube: *P *= 2.61 × 10^−7^; 5-tube: *P *= 2.66 × 10^−5^). In contrast, eugenol exhibited comparatively stable flying responses across the 1-, 3-, and 5-active-tube conditions, with minimal temporal restructuring. These results indicate that flying represented an exposure-level-sensitive activity response with treatment-specific temporal reorganization rather than a uniform, exposure-level-dependent increase.

Walking activity showed no detectable response to treatment identity, active tube exposure level, or time (Fig. 3C). Neither treatment identity (*F*_1,134.41_ = 3.06, *P *= 0.083), time (*F*_1,132_ = 0.01, *P *= 0.919), nor active tube exposure level (*P *= 0.318) significantly influenced walking, and all interaction terms were nonsignificant (*P *> 0.23). Although peppermint oil produced slightly higher walking levels, these differences were small and not statistically supported. Walking therefore remained the most stable and least responsive to changes in active tube exposure level, consistent with its narrow dynamic range throughout the assay. Consistent with the exposure level-dependent escape and flight activity responses, vertical occupancy within the release cage also varied over time across the 1, 3, and 5 active tube conditions, with eugenol and peppermint oil differing across 1-, 3-, and 5-tube deployments in upper- versus lower-cage occupancy trajectories over the assay period (Fig. S2).

### Essential Oil Mixtures Produce Distinct and Behavior-Specific Patterns in Escape, Flying, and Walking

Escape responses differed primarily in magnitude rather than in statistically distinct temporal trajectories (Fig. 4A). Escape magnitude increased over time across both mixtures (*F*_1,42_ = 7.42, *P *= 0.0094). The eugenol-dominant mixture (eugenol:peppermint oil 3:1) elicited consistently higher escape proportions across all time points relative to the peppermint oil-dominant mixture (eugenol:peppermint oil 1:3), although this difference did not reach statistical significance (*F*_1,42_ = 2.40, *P *= 0.129). Visually, both mixtures exhibited modest upward trajectories over the assay period, with the 3:1 mixture increasing slightly from an elevated baseline and the 1:3 mixture showing a more gradual rise from lower initial escape levels. However, the mixture-by-time interaction was not statistically significant (*F*_1,42_ = 2.15, *P *= 0.150), indicating that blending the oils did not generate detectably different temporal escape dynamics. The relative escape profiles of the 2 mixtures were broadly consistent with those of the more strongly represented component, although the experiment was not designed to test formal additive or non-additive interactions (Fig. 3A).

**Fig. 4.**
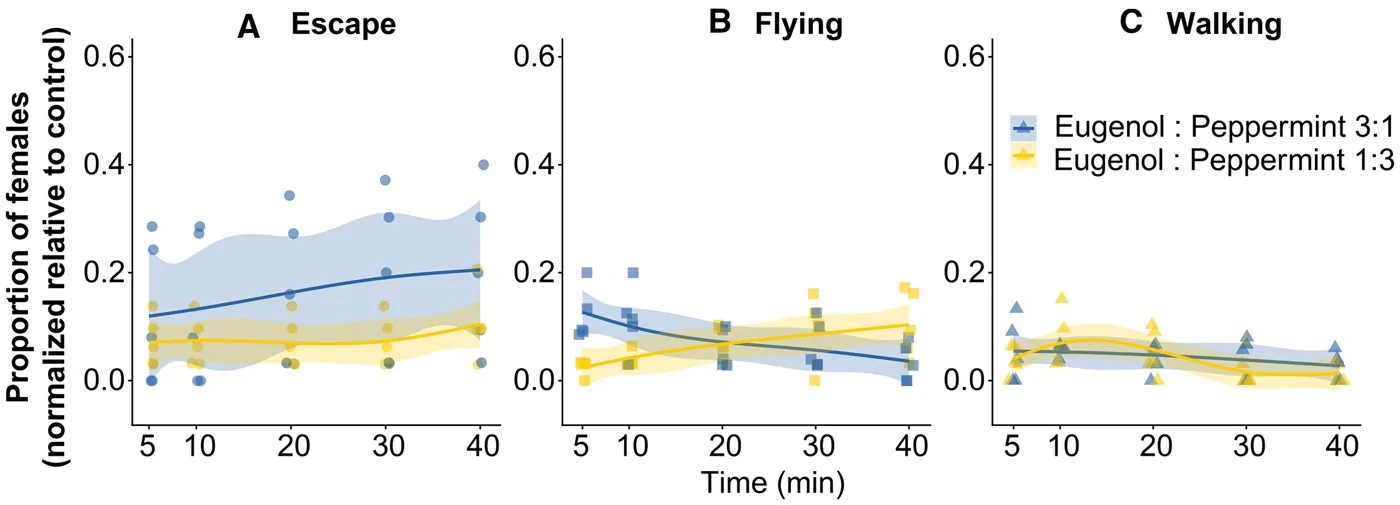
Temporal dynamics of escape, flying, and walking behavior under exposure to eugenol–peppermint oil mixtures. Temporal trajectories of *Cx. quinquefasciatus* escape (4A), flying (4B), and walking (4C) responses are shown over a 40-min assay for 2 active tube mixtures of eugenol and peppermint oil (3:1 and 1:3 blends). Each point represents the proportion of mosquitoes exhibiting the indicated behavior within an independent trial at 5, 10, 20, 30, or 40 min, expressed as the treatment response minus the corresponding response in the paired mineral oil control at the same observation time, with zero indicating no increase relative to the control. Behavioral responses are distinguished by shape (circles = escape, squares = flying, triangles = walking), and mixture treatments are indicated by color (blue = eugenol + peppermint oil 3:1; yellow = eugenol + peppermint oil 1:3). Solid lines show model-based natural-spline smooths (df = 3), with shaded ribbons denoting the 95% confidence interval of the fit. Each assay included 25 to 35 female mosquitoes. Five independent trials were conducted for each mixture treatment. Because the same replicate was sampled repeatedly over time, time was treated as a repeated-measures factor in mixed-effects analyses, with mixture composition included as a fixed effect.

Flight activity showed the clearest mixture-specific divergence (Fig. 4B). The eugenol-dominant 3:1 mixture elicited elevated early flying followed by a steady decline across the assay period (*β* = −0.00246 ± 0.00062). In contrast, the peppermint oil-dominant 1:3 mixture did not elevate early flying relative to the mineral oil control (*t *= −5.18, *P *= 5.95 × 10^−6^), but flying activity increased progressively over time (*β* = +0.00469 ± 0.00088). The pronounced mixture-by-time interaction (*F*_1,42_ = 28.38, *P *= 3.66 × 10^−6^) shows that the 2 mixtures follow sharply divergent temporal trajectories. Notably, there was no overall main effect of time (*F*_1,42_ = 0.07, *P *= 0.790), indicating that the opposing slopes of the 2 mixtures offset one another when averaged. These dynamics mirror the patterns observed for the dominant individual oils, with peppermint oil-associated flight response increasing over time and eugenol-associated flight response declining over time, indicating that the temporal flight activity profiles of the mixtures more closely resembled those associated with the more strongly represented component than a simple intermediate pattern.

Walking activity remained comparatively stable and minimally affected under exposure to either essential oil mixture (Fig. 4C). Neither mixture identity (*F*_1,42_ = 0.12, *P *= 0.735) nor its interaction with time (*F*_1,42_ = 0.47, *P *= 0.498) significantly altered walking behavior. A modest overall decline across the assay period (*F*_1,42_ = 7.59, *P *= 0.0086) was observed in both mixtures. Although the peppermint oil-dominant mixture exhibited a slight early elevation in walking before gradually declining, visually resembling the peppermint oil-only treatment, the magnitude of this effect was small, confidence intervals overlapped broadly, and temporal slopes did not differ statistically between mixtures. Thus, walking remained weakly responsive relative to escape and flight activity, and mixture composition did not produce robust, behavior-specific modulation of walking activity. Vertical occupancy within the release cage showed mixture-specific temporal patterns that appeared broadly consistent with the flight responses of the eugenol:peppermint oil mixtures, suggesting that increased flight activity may have contributed to greater upper-cage occupancy during the assay period (Fig. S3).

### Joint Escape–Activity Trajectories Show Treatment-Specific Temporal Organization

Plotting model-estimated control-adjusted escape and flight activity in a common response space showed visually distinct treatment-specific trajectories that were consistent with the endpoint-specific mixed-effects analyses (Fig. 5). Eugenol exhibited comparatively high escape from the earliest observation and remained at a similar escape level throughout the assay, while flight activity declined overall. This compact trajectory shows that most of the displacement response was established early and subsequently remained stable. Citronellol and geraniol moved progressively upward and leftward, reflecting increasing escape and declining flight activity over time.

**Fig. 5.**
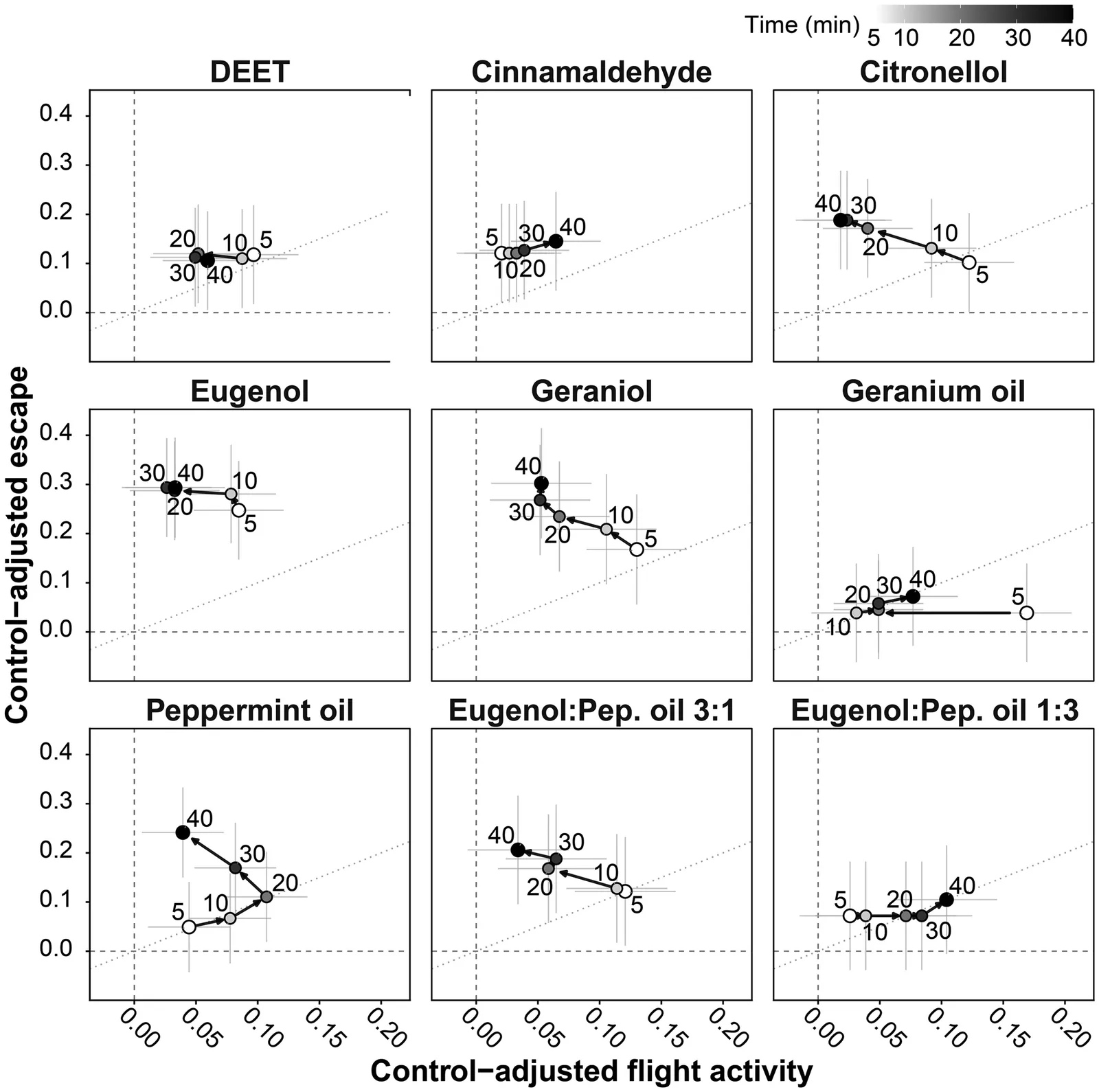
Joint temporal trajectories of control-adjusted escape and flight activity under volatile treatments and botanical mixtures. Control-adjusted flight activity is plotted against control-adjusted escape for DEET, cinnamaldehyde, citronellol, eugenol, geraniol, geranium oil, peppermint oil, and the eugenol:peppermint oil 3:1 and 1:3 mixtures. Each point represents the model-estimated marginal mean at 5, 10, 20, 30, or 40 min. Points are connected chronologically within each treatment, and arrows indicate the direction of movement through the joint response space as the assay progressed. Point shading becomes darker with increasing observation time. Horizontal and vertical error bars represent marginal 95% confidence intervals for flight activity and escape, respectively. Dashed horizontal and vertical reference lines indicate no difference from the paired mineral-oil control. The dotted diagonal indicates equal numerical magnitudes of control-adjusted escape and flight activity; it does not represent equal behavioral contribution or a physiological tradeoff. Movement upward indicates increasing escape, movement to the right indicates increasing flight activity, and movement upward and to the left indicates increasing escape accompanied by declining flight activity. Individual-treatment assays included 23 to 40 female mosquitoes and were represented by 5 independent trials per treatment, except geraniol, which was represented by 4 trials, and peppermint oil, which was represented by 6 trials. Each mixture assay included 25 to 35 female mosquitoes, with 5 independent trials per mixture. Individual treatments were evaluated using 5 active tubes, whereas each mixture contained 4 active tubes arranged in the indicated 3:1 or 1:3 ratio. The trajectories provide an integrative visualization of the 2 behavioral endpoints; statistical inference was based on the corresponding longitudinal mixed-effects models.

Peppermint oil exhibited the greatest temporal reorganization. Escape and flight activity increased together through approximately 20 min, after which escape continued to increase while flight activity declined. At the cohort level, this pattern is consistent with greater early within-chamber activation followed by delayed displacement. Importantly, it does not demonstrate that individual mosquitoes recorded flying subsequently escaped. Geranium oil began with comparatively high flight activity and low escape, followed by a marked reduction in flight activity and only a modest increase in escape. Cinnamaldehyde and DEET followed comparatively compact trajectories with moderate responses and less temporal restructuring.

The 2 mixture treatments also differed in their temporal organization. The eugenol-rich 3:1 mixture shifted overall toward greater escape and lower flight activity, whereas the peppermint-rich 1:3 mixture shifted primarily toward greater flight activity through 30 min and showed only a modest increase in escape at 40 min. Thus, the mixtures retained component-associated directional tendencies but differed in response magnitude and temporal form from the corresponding individual treatments. Because the individual-treatment and mixture assays used different active-tube configurations, comparisons among assay series are descriptive. The trajectory plot was used as an integrative visualization, while statistical inference remained based on the corresponding endpoint-specific mixed-effects models.

### Sewer-Water Volatiles Attenuate Behavioral Responses But Preserve Relative Treatment

Physicochemical characterization of pooled USDS water revealed a mildly circumneutral pH (6.83), electrical conductivity of 710 µS cm^−^^1^, total organic carbon (62.61 ppm), and total nitrogen (7.791 ppm). The conductivity indicates moderate dissolved ionic strength, and the elevated TOC and TN values indicate a chemically enriched aqueous matrix with substantial organic and nitrogenous inputs. Escape responses under exposure to USDS-water condition differed significantly between treatments. The eugenol:peppermint oil 3:1 mixture produced greater escape than eugenol alone (*F*_1,42_ = 4.62, *P *= 0.037; Fig. 6A). Escape did not change significantly over time (*F*_1,42_ = 1.68, *P *= 0.202), and the treatment-by-time interaction was not significant (*F*_1,42_ = 0.03, *P *= 0.856), indicating that the temporal patterns were broadly similar between treatments. When assays conducted with and without USDS water were compared, the magnitude of the difference in escape varied significantly between treatments (*F*_1,36_ = 7.78, *P *= 0.0084; Fig. 6A, lower panel), with the presence of USDS water associated with a greater reduction in escape response for the mixture than for eugenol.

**Fig. 6.**
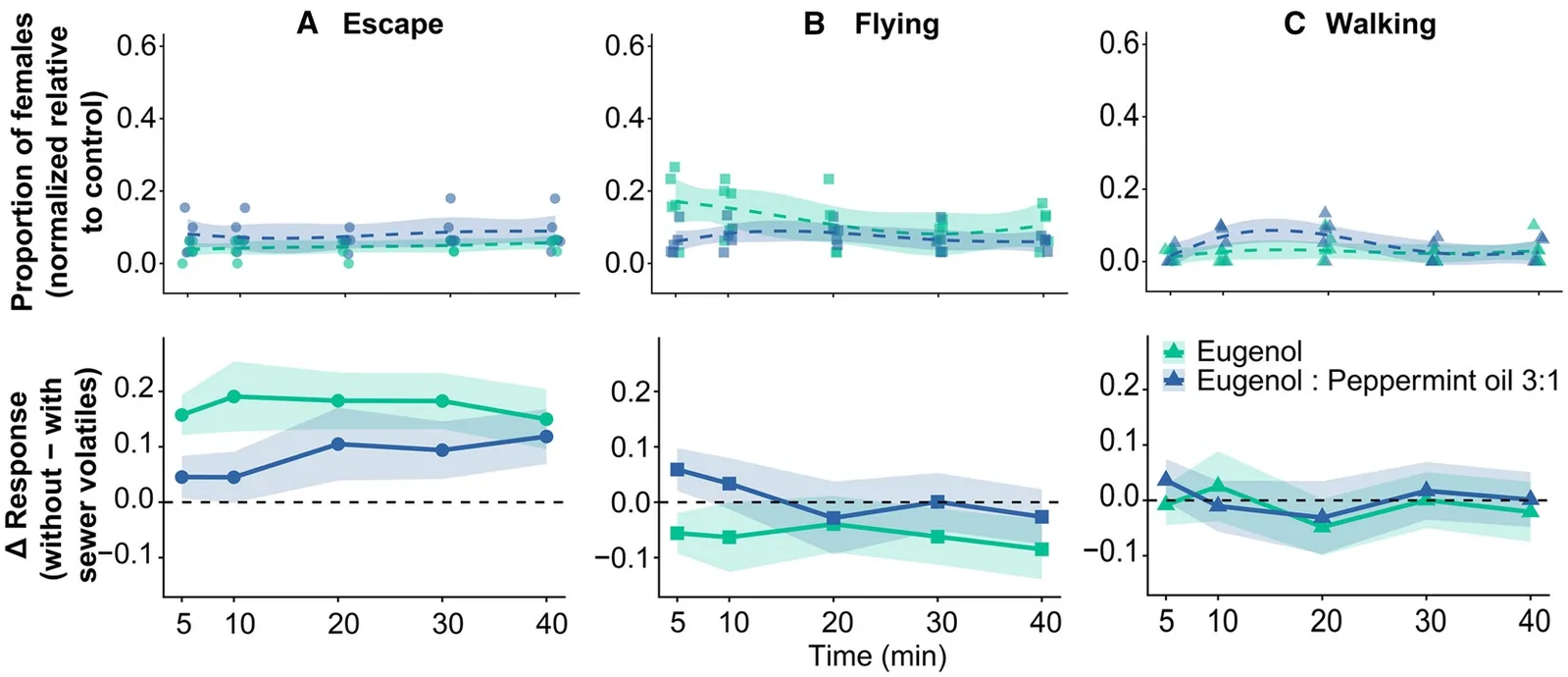
Behavioral responses to eugenol and a eugenol–peppermint oil (3:1) mixture in the presence and absence of USDS-water volatiles. (Top panels) Escape (A), flight activity (B), and walking (C) by *Culex quinquefasciatus* during 40-min exposures to eugenol or the eugenol:peppermint oil 3:1 mixture in the presence of USDS water. Eugenol was delivered from 3 active eugenol tubes, whereas the mixture comprised 3 active eugenol tubes and 1 active peppermint-oil tube. Each point represents the control-adjusted proportion of females exhibiting the indicated behavior within an independent trial at 5, 10, 20, 30, or 40 min, calculated by subtracting the response in the corresponding paired mineral-oil control at the same observation time. Behavioral endpoints are distinguished by symbol: circles indicate escape, squares indicate flight activity, and triangles indicate walking. Lines show natural-spline fits (df = 3), and shaded bands represent 95% confidence intervals. Zero, represented by the lower axis boundary, indicates no difference from the paired control. (Bottom panels) The between-condition difference for each treatment, calculated as the response in assays conducted without USDS water minus the corresponding response in the presence of USDS water. Positive values indicate a larger response without USDS water, negative values indicate a larger response in the presence of USDS water, and zero indicates no difference between conditions. The dashed horizontal line denotes zero. Eugenol assays included 30 to 33 females per assay, whereas mixture assays included 30 to 39 females per assay. Each treatment was represented by 5 independent trials, with the same cohort observed repeatedly at all 5 time points. Mixed-effects analyses identified overall differences between eugenol and the mixture under the USDS-water condition for escape (*F*_1,42_ = 4.62, *P *= 0.037) and flight activity (*F*_1,42_ = 10.78, *P *= 0.002). The magnitude of the between-condition difference also varied between treatments for escape (*F*_1,36_ = 7.78, *P *= 0.0084) and flight activity (*F*_1,37_ = 8.35, *P *= 0.0064). These statistics represent overall treatment effects across the repeated-measures assay rather than pairwise comparisons at individual time points; accordingly, time-specific significance annotations are not shown.

Flight activity under the USDS-water condition was significantly lower for the eugenol:peppermint oil 3:1 mixture than for eugenol alone (*F*_1,42_ = 10.78, *P *= 0.002; Fig. 6B). Flight activity declined over time (*F*_1,42_ = 4.68, *P *= 0.036), whereas the treatment-by-time interaction was not significant (*F*_1,42_ = 2.75, *P *= 0.104), indicating no detectable difference in the temporal pattern between treatments. The magnitude of the difference between assays conducted with and without USDS water also varied significantly between treatments (*F*_1,37_ = 8.35, *P *= 0.0064; Fig. 6B, lower panel), with a larger reduction in flight activity under the USDS-water condition for the mixture than for eugenol.

Walking activity remained low under the USDS-water condition and did not differ significantly between treatments (*F*_1,42_ = 3.00, *P *= 0.091; Fig. 6C). Walking also showed no significant change over time (*F*_1,42_ = 0.05, *P *= 0.821) and no significant treatment-by-time interaction (*F*_1,42_ = 0.97, *P *= 0.330). The magnitude of the difference between assays conducted with and without USDS water did not differ between treatments (*F*_1,37_ = 0.06, *P *= 0.816; Fig. 6C, lower panel). Walking therefore remained the least responsive of the 3 behavioral measures. Complementary vertical-occupancy analyses showed declining upper-cage occupancy and increasing lower-cage occupancy over time for both eugenol and the eugenol:peppermint oil 3:1 mixture under the USDS-water condition (Fig. S4).

### Post-Exposure Mortality

Twenty-four hour mortality data were available for 20 of 23 mineral-oil control trials and for varying proportions of the analyzed treatment trials (Table S3). Mean trial-level mortality was low under most conditions, including mineral-oil controls (1.29 ± 0.55%), but was elevated following 5-active-tube exposures to peppermint oil (15.15 ± 7.45%) and DEET (10.80 ± 6.46%). The highest mortality occurred following exposure to the eugenol:peppermint oil 3:1 mixture in the presence of USDS-water volatiles (34.91 ± 6.63%), compared with 5.10 ± 2.71% for the same mixture without USDS-water volatiles. No 24-h mortality observations were available for the 5-active-tube eugenol or geraniol trials. Treatment-specific means, standard errors, and data availability are reported in Table S3. Trials lacking recorded mortality observations were treated as missing and excluded from the corresponding calculations.

## Discussion

This study demonstrates that essential oils, their mixtures, and sewer-derived volatiles each influence *Cx. quinquefasciatus* behavior in multiple measurable ways rather than producing a single uniform repellency effect. By jointly examining multiple behavioral response modalities (escape, flight, and walking), we observed a spectrum of responses expressed along 2 operationally distinct behavioral dimensions: mosquito escape, which represented spatial displacement from the treated chamber, and within-chamber behavioral activation, expressed as flight or walking among mosquitoes that had not departed. These behavioral patterns were observed across active tube exposure levels, mixture treatments, and odor backgrounds, indicating that responses varied systematically among treatments rather than reflecting undifferentiated activity. Although these patterns represent short-term activity within a controlled assay, they offer a practical way to compare volatile repellents and extend existing frameworks that view spatial repellency as a multidimensional process rather than a single endpoint (Norris and Coats 2017, Achee and Grieco 2022).

The principal methodological contribution of this study is therefore not the initial demonstration that botanical volatiles alter mosquito behavior, but the use of a single repeated-measures assay to distinguish several components of that response. Recording escape, flying, walking, and vertical position at multiple observation times allowed the evaluation of treatment-induced departure from the chamber separately from behavioral activation among mosquitoes that remained inside. Applying this framework across individual compounds, whole essential oils, vapor-phase mixtures, active-tube exposure levels, and a USDS-water odor background provided a consistent basis for comparing treatment-specific behavioral profiles under increasingly complex exposure conditions.

The botanical volatile treatments displayed distinct behavioral profiles that differed in both the magnitude and temporal expression of escape and within-chamber activity. Individual compounds such as eugenol, geraniol, and citronellol primarily elicited an escape response with limited activity-based disturbance, consistent with classical spatial repellency observed in earlier chamber and arm-in-cage studies (Mohd-Zaki et al. 2005, Hao et al. 2014, Farooq et al. 2022, Lopez et al. 2025). These escape-oriented responses remained recognizable in the mixture treatments and in the presence of sewer-water odors. Although our study did not investigate underlying physiological pathways, this stability suggests that these oils reliably trigger displacement-type behaviors across a range of conditions. Operationally, such profiles align with tools designed to reduce mosquito entry or indoor resting through use of airborne spatial repellent delivery systems, including passive emanators and other volatile-release devices (World Health Organization 2013, Norris and Coats 2017). The joint trajectories further demonstrate that these responses were not fixed behavioral categories. Eugenol elicited an early escape response that plateaued after approximately 20 min, whereas citronellol and geraniol showed a more gradual shift toward escape as flight activity declined. Peppermint oil followed a more dynamic pathway, with greater early within-chamber activation (disturbance) followed by increasing escape (displacement) later in the assay. Treatment differences therefore involved not only the magnitude of escape and flight activity but also the timing with which the 2 responses were expressed.

Peppermint oil and the peppermint oil-rich mixture produced a contrasting behavioral pattern characterized primarily by changes in flight activity, whereas walking activity remained comparatively stable and early escape was limited. This pattern indicates a relatively specific activation of flight rather than generalized locomotor stimulation. Similar increases in activity and disruption of resting have been described in other excito-repellency studies (Sathantriphop et al. 2014a, 2014b). However, because flight activity was recorded among mosquitoes that remained within the release cage, increased flight did not independently demonstrate directed movement away from the volatile source and was therefore interpreted as behavioral activation or disturbance rather than direct evidence of repellency. Peppermint oil also showed exposure-level-dependent changes in flight activity, shifting from mild suppression under the 1-active-tube condition to pronounced activation under the 3- and 5-active-tube conditions. Whether this flight activation subsequently promotes departure, disrupts resting, or reduces residence time in larger enclosed habitats cannot be determined from the present assay and requires direct evaluation under semi-field and field conditions (Contreras-Perera et al. 2022).

The mixture experiments showed that behavioral responses varied with the relative representation of eugenol and peppermint oil among the active treatment tubes. The eugenol-rich 3:1 treatment generally produced a stronger escape-oriented profile, whereas the peppermint oil-rich 1:3 treatment showed a temporal flight activity pattern more similar to that observed with peppermint oil alone. Thus, the mixture responses tended to resemble the behavioral tendencies associated with the more strongly represented component rather than consistently occupying an intermediate position between the individual treatments. Previous studies have shown that behavioral responses to volatile blends may not be readily predicted from the effects of their individual components, particularly when the components differ in volatility and in their relative contributions to the resulting odor profile (Peach et al. 2019, Afify and Potter 2020). However, the patterns observed here should not be interpreted as evidence of additivity, synergy, or antagonism because airborne concentrations and component-specific emission rates were not quantified, and the experiment did not include component treatments matched by active tube exposure level or an explicit additive null model. The findings therefore support empirical evaluation of volatile mixtures while underscoring that formal characterization of mixture interactions will require quantified vapor-phase exposures and predictions derived from component-specific exposure–response relationships.

The influence of sewer-water volatiles highlights how odor-rich environments modulate repellent-driven behavior. The magnitudes of escape and within-chamber flight activity were reduced in the presence of VOCs, aligning with evidence that background odors can mask or dilute behavioral responses to volatiles (Achee et al. 2012, Afify and Potter 2020). Walking activity remained weakly responsive across conditions and did not show the same clear reduction. Physicochemical characterization of the pooled USDS water indicated moderate ionic content and substantial organic and nitrogenous loading relative to many surface freshwater systems, consistent with nitrogen-enriched urban drainage infrastructure (Paul and Meyer 2001, Wetzel 2001, Walsh et al. 2005, Kaushal et al. 2018). Elevated organic carbon and nitrogen loads in enclosed drainage systems are commonly associated with microbial activity, organic debris accumulation, and intermittent anoxic microsites, conditions that facilitate microbial metabolism and organic matter degradation (Withers et al. 2014). Such nutrient-rich, organically enriched environments are also characteristic of productive larval habitats for *Cx. quinquefasciatus*, which is strongly associated with polluted urban water systems (Lund et al. 2014). These processes can generate chemically diverse volatile emissions, including short-chain fatty acids, sulfur-containing compounds, and other low-molecular-weight microbial byproducts that contribute to complex headspace chemistry. One plausible explanation is that sewer-derived VOCs reduce the salience of essential oil cues, either through competitive odor interference or by shifting behavioral context toward reduced activity or increased resting. Although the present assays did not directly quantify microbial activity or resolve sensory mechanisms, the chemical profile of the USDS water suggests a biogeochemically active matrix capable of producing volatiles that may interact with plant-derived odorants. This interpretation is consistent with prior work showing that complex odor backgrounds can dampen volatile-driven mosquito behaviors (McBride et al. 2014, Afify and Potter 2020). The elevated 24-h mortality observed under selected conditions, particularly for the eugenol:peppermint oil 3:1 mixture in the presence of sewer-water volatiles, indicates that some exposures produced delayed toxic effects in addition to the behavioral responses recorded during the assay. Because mortality was assessed after completion of the 40-min behavioral assay, its contribution to the observed behavioral patterns cannot be determined directly. Even though 24-h mortality was low for most treatment conditions, suggesting little impact on behavioral response outcomes, these findings warrant caution in attributing the responses under these conditions exclusively to nonlethal behavioral modification. Importantly, however, the relative ordering among essential oils and essential oil mixtures was preserved. This indicates that while environmental odors reduced the magnitude of the measured behavioral responses, they did not fundamentally alter the qualitative behavioral profiles induced by different odor sources. Since *Cx. quinquefasciatus* commonly occupies chemically complex habitats such as storm drains, catch basins, and wastewater systems, incorporating environmental odors into laboratory evaluations may improve relevance to field settings. Nevertheless, sewer-water VOCs represent only 1 odor environment, and VOC composition is likely to vary among environments or even within a single environment. Therefore, broader environmental sampling, paired with chemical profiling and behavioral validation, would help determine the generalizability of these attenuation effects.

The USDS application considered here differs from the traditional use of botanical repellents as personal protectants. In storm drains, the operational objective would be to alter mosquito use of an enclosed habitat rather than prevent landing or biting on an individual. Treatments that consistently promote departure from a treated chamber may be candidates for reducing adult retention within protected resting sites, whereas treatments that increase flight without producing departure may indicate disturbance but cannot, on their own, be assumed to reduce occupancy. Increased flight activity could plausibly interrupt resting or increase the probability of encountering an exit, but neither mechanism was directly tested here. Consequently, flight activation should be considered a candidate indicator of habitat disturbance rather than evidence for repellency or reduced site occupancy. The persistence of treatment-specific response patterns in the presence of USDS-water odors supports further evaluation under semi-field and field conditions that directly measure habitat use, including entry, residence time, departure, and reentry. Such studies will be necessary to determine whether repeated or sustained exposure to volatile compounds can complement existing larval and adult mosquito-control strategies in underground drainage systems.

Adult mosquito populations within USDS include individuals representing a wide range of ages, sexes, mating states, and gonotrophic conditions, reflecting the multiple roles of these structures as sites of adult emergence, resting, refuge, and proximity to aquatic developmental habitats (Strickman and Lang 1986, Reisen et al. 1990, Wang et al. 2021). We focused on young nulliparous females as a manageable and operationally important cohort. These females have not yet completed a blood meal or contributed to egg production and therefore represent a critical stage linking adult emergence to subsequent host seeking, biting, and population recruitment. In the hot, arid communities of southern California, underground storm drains can provide protected resting refuges from otherwise less hospitable outdoor conditions (Strickman and Lang 1986, Popko et al. 2018). Volatile treatments that discourage young females from entering or remaining in these refuges, or that promote their departure, could increase their exposure to unfavorable environmental conditions and thereby reduce survival, biting pressure, and subsequent reproductive output. These broader population-level effects were not measured directly in the present study. Because the present study focused on young nulliparous females, future experiments should also determine whether mosquitoes in other physiological states, particularly blood-fed and gravid females, respond similarly under more realistic USDS conditions, as physiological condition can influence mosquito motivation, movement, resting behavior, host seeking, and habitat use (Hoi and Roitberg 2014). Assays using USDS water as an oviposition substrate and directly quantifying oviposition would provide a more direct test of whether volatile-induced disturbance alters mosquito use of USDS as reproductive habitat.

Several caveats should be considered. Our assays measured short-term responses within a confined chamber and may not fully represent mosquito behavior in larger, more open, or operational environments. The 3 behavioral metrics, although informative, do not encompass all potential forms of repellency, avoidance, or behavioral disturbance. Connecting-tube entry and arrival in the escape cage represented successive stages of movement; however, tube occupancy was transient and consistently progressed toward the escape cage, with no observed return to the release chamber. The analysis therefore focused on cumulative departure from the treated chamber rather than the time required to complete movement through the escape pathway. Because mosquitoes classified as escaped were no longer scored for activity within the release cage, escape and flight represent complementary cohort-level outcomes rather than fully independent behavioral responses. Accordingly, their opposing patterns should be interpreted as differences in how treatments distributed mosquitoes between departure from the chamber and flight activity among those remaining, rather than as evidence of an intrinsic physiological tradeoff. Finally, the mixture and odor-interference experiments were evaluated under specific nominal exposure and airflow conditions that may differ from those encountered in operational settings. These limitations do not diminish the comparative value of the assay but identify areas where individual tracking, longer observation periods, semi-field experiments, and quantitative characterization of airborne volatiles would strengthen future evaluations.

Together, these findings support a behavioral framework that distinguishes departure from a treated space from activity responses occurring among mosquitoes that remain within it, consistent with broader interpretations of spatial repellency as a multidimensional process (Norris and Coats 2017, Achee and Grieco 2022). Treatments that produced rapid and sustained escape, particularly eugenol, may warrant further evaluation as area-based behavioral interventions in enclosed habitats such as USDS, where altering mosquito microhabitat use could complement conventional control approaches (Hoi and Roitberg 2014, Norris and Coats 2017). In contrast, peppermint oil primarily altered flight activity, indicating clear behavioral detection and disturbance but not necessarily departure from, or reduced occupancy of, the treated environment. The persistence of these treatment-specific profiles in the presence of USDS-water odors suggests that botanical volatiles remain behaviorally detectable under chemically complex conditions, although the magnitude of response may be attenuated. Future semi-field and field experiments should determine whether these short-term responses translate into reduced entry, resting duration, retention, reentry, or oviposition within underground drainage systems. Such studies are needed to establish whether botanical volatiles can complement existing mosquito-control approaches in urban infrastructures where restricted access, organic contamination, and insecticide resistance constrain conventional interventions (Hoi and Roitberg 2014, Norris and Coats 2017, Afify and Potter 2020).

## Supplementary Material

tjag168_Supplementary_Data

## Data Availability

Data from this study are available from the Dryad Digital Repository (Chandrasegaran et al. 2026) at https://doi.org/10.5061/dryad.bk3j9kdtf.

## References

[tjag168-B1] Achee NL , Bangs MJ, Farlow R, et al 2012. Spatial repellents: from discovery and development to evidence-based validation. Malar. J. 11:164. 10.1186/1475-2875-11-164 PMC3453515 22583679

[tjag168-B2] Achee NL , Grieco JP. 2018. Current evidence, new insights, challenges and future outlooks to the use of spatial repellents for public health. In: *ACS symposium series.* American Chemical Society. p. 25–42. 10.1021/bk-2018-1289.ch003

[tjag168-B3] Achee NL , Grieco JP. 2022. Current status of spatial repellents in the global vector control community. In: *Advances in arthropod repellents*. p. 267–278. 10.1016/B978-0-323-85411-5.00009-1

[tjag168-B4] Achee NL , Perkins TA, Moore SM, et al 2023. Spatial repellents: the current roadmap to global recommendation of spatial repellents for public health use. Curr. Res. Parasitol. Vector. Borne Dis. 3:100107. 10.1016/j.crpvbd.2022.100107 PMC9801085 36590345

[tjag168-B5] Afify A , Betz JF, Riabinina O, et al 2019. Commonly used insect repellents hide human odors from *Anopheles* mosquitoes. Curr. Biol. 29:3669–3680.e5. 10.1016/j.cub.2019.09.007 PMC6832857 31630950

[tjag168-B6] Afify A , Potter CJ. 2020. Insect repellents mediate species-specific olfactory behaviours in mosquitoes. Malar. J. 19:127. 10.1186/s12936-020-03206-8 PMC7106743 32228701

[tjag168-B7] Ansari MA , Vasudevan P, Tandon M, et al 2000. Larvicidal and mosquito repellent action of peppermint (*Mentha piperita*) oil. Bioresour. Technol. 71:267–271. 10.1016/S0960-8524(99)00079-6

[tjag168-B8] Bisset JA , Rodriguez MM, Diaz C, et al 1990. The mechanisms of organophosphate and carbamate resistance in *Culex quinquefasciatus* (Diptera: Culicidae) from Cuba. Bull. Entomol. Res. 80:245–250. 10.1017/S0007485300050434

[tjag168-B9] Boonyuan W , Grieco JP, Bangs MJ, et al 2014. Excito-repellency of essential oils against an *Aedes aegypti* field population in Thailand. J. Vector Ecol. 39:112–122. 10.1111/j.1948-7134.2014.12077.x 24820563

[tjag168-B10] Chandrasegaran K , et al 2026. Data from: multidimensional behavioral signatures of Culex quinquefasciatus in response to botanical volatiles reveal divergent patterns of mosquito displacement and behavioral disturbance [Dataset]. Dryad Digital Repository. 10.5061/dryad.bk3j9kdtf PMC13743431 42995421

[tjag168-B11] Contreras-Perera J , Martin-Park A, Puerta-Guardo H, et al 2022. Mosquito excito-repellency: effects on behavior and the development of insecticide resistance. Mosquito Res. 12:3. 10.5772/intechopen.105755

[tjag168-B12] Deletre E , Chandre F, Williams L, et al 2015. Electrophysiological and behavioral characterization of bioactive compounds of the *Thymus vulgaris*, *Cymbopogon winterianus*, *Cuminum cyminum* and *Cinnamomum zeylanicum* essential oils against *Anopheles gambiae* and prospects for their use as bednet treatments. Parasit. Vectors. 8:316. 10.1186/s13071-015-0934-y PMC4470088 26063119

[tjag168-B13] Des Lauriers A , Li J, Sze K, et al 2006. A field study of the use of methoprene for West Nile virus mosquito control. J. Environ. Eng. Sci. 5:517–527. 10.1139/s06-018

[tjag168-B14] Dethier VG , Browne BL, Smith CN. 1960. The designation of chemicals in terms of the responses they elicit from insects. J. Econ. Entomol. 53:134–136. 10.1093/jee/53.1.134 20069831

[tjag168-B15] Estrada JG , Mulla MS. 1986. Evaluation of two new insect growth regulators against mosquitoes in the laboratory. J. Am. Mosq. Control Assoc. 2:57–60. 2906962

[tjag168-B16] Farooq M , Bangonan L, Xue RD, et al 2022. Evaluation of essential oils as spatial repellents against *Aedes aegypti* in an olfactometer. J. Am. Mosq. Control Assoc. 38:261–267. 10.2987/22-7090 36351363

[tjag168-B17] Hao H , Wei J, Dai J, et al 2014. Host-seeking and blood-feeding behavior of *Aedes albopictus* (Diptera: Culicidae) exposed to vapors of geraniol, citral, citronellal, eugenol, or anisaldehyde. J. Med. Entomol. 45:533–539. 10.1093/jmedent/45.3.533 18533449

[tjag168-B18] Hoi AG , Roitberg BD. 2014. Mosquito behaviour and disease control. Evol. Med. Public Health. 2014:162. 10.1093/emph/eou030 PMC4268418 25432163

[tjag168-B19] Kaushal SS , Gold AJ, Bernal S, et al 2018. Watershed “chemical cocktails”: forming novel elemental combinations in Anthropocene fresh waters. Biogeochemistry. 141:281–305. 10.1007/s10533-018-0502-6 PMC6699637 31427837

[tjag168-B20] Lopez AD , Whyms S, Luker HA, et al 2025. Repellency of essential oils and plant-derived compounds against *Aedes aegypti*. Insects. 16:51. 10.3390/insects16010051 PMC11765945 39859632

[tjag168-B21] Low VL , Chen CD, Lee HL, et al 2013. Enzymatic characterization of insecticide resistance mechanisms in field populations of Malaysian *Culex quinquefasciatus* Say (Diptera: Culicidae). PLoS One. 8:e79928. 10.1371/journal.pone.0079928 PMC3836847 24278220

[tjag168-B22] Lund A , McMillan J, Kelly R, et al 2014. Long term impacts of combined sewer overflow remediation on water quality and population dynamics of *Culex quinquefasciatus*, the main urban West Nile virus vector in Atlanta, GA. Environ. Res. 129:20–26. 10.1016/j.envres.2013.12.008 24528998

[tjag168-B23] McBride CS , Baier F, Omondi AB, et al 2014. Evolution of mosquito preference for humans linked to an odorant receptor. Nature. 515:222–227. 10.1038/nature13964 PMC4286346 25391959

[tjag168-B24] Mohd-Zaki Z , Mohd Ali NA, Ahmad S, et al 2005. Four monoterpene compounds: repellency evaluation against *Aedes aegypti*. Malays. J. Sci. 24:225–228.

[tjag168-B25] Nararak J , Sathantriphop S, Kongmee M, et al 2019. Excito-repellent activity of β-caryophyllene oxide against *Aedes aegypti* and *Anopheles minimus*. Acta Trop. 197:105030. 10.1016/j.actatropica.2019.05.021 31121148

[tjag168-B26] Nerio LS , Olivero-Verbel J, Stashenko E. 2010. Repellent activity of essential oils: a review. Bioresour. Technol. 101:372–378. 10.1016/j.biortech.2009.07.048 19729299

[tjag168-B27] Noosidum A , Prabaripai A, Chareonviriyaphap T, et al 2008. Excito-repellency properties of essential oils from *Melaleuca leucadendron* L., *Litsea cubeba* (Lour.) Persoon, and *Litsea salicifolia* (Nees) on *Aedes aegypti* (L.) mosquitoes. J. Vector Ecol. 33:305–312. 10.3376/1081-1710-33.2.305 19263850

[tjag168-B28] Norris EJ , Coats JR. 2017. Current and future repellent technologies: the potential of spatial repellents and their place in mosquito-borne disease control. IJERPH. 14:124. 10.3390/ijerph14020124 PMC5334678 28146066

[tjag168-B29] Paul MJ , Meyer JL. 2001. Streams in the urban landscape. Annu. Rev. Ecol. Syst. 32:333–365. 10.1146/annurev.ecolsys.32.081501.114040

[tjag168-B30] Peach DAH , Almond M, Gries R, et al 2019. Lemongrass and cinnamon bark: plant essential oil blend as a spatial repellent for mosquitoes in a field setting. J. Med. Entomol. 56:1346–1352. 10.1093/jme/tjz078 31143941

[tjag168-B31] Peiris HTR , Hemingway J. 1993. Characterization and inheritance of elevated esterases in organophosphorus and carbamate insecticide resistant *Culex quinquefasciatus* (Diptera: Culicidae) from Sri Lanka. Bull. Entomol. Res. 83:127–132. 10.1017/S0007485300041869

[tjag168-B32] Popescu IE , Gostin IN, Blidar CF. 2024. An overview of the mechanisms of action and administration technologies of the essential oils used as green insecticides. AgriEngineering. 6:1195–1217. 10.3390/agriengineering6020068

[tjag168-B33] Popko DA , Henke JA, Mullens BA, et al 2018. Evaluation of two entomopathogenic fungi for control of *Culex quinquefasciatus* (Diptera: Culicidae) in underground storm drains in the Coachella Valley, California, United States. J. Med. Entomol. 55:654–665. 10.1093/jme/tjx233 29294059

[tjag168-B34] Reisen WK , Meyer RP, Tempelis CH, et al 1990. Mosquito abundance and bionomics in residential communities in Orange and Los Angeles Counties, California. J. Med. Entomol. 27:356–367. 10.1093/jmedent/27.3.356 1970608

[tjag168-B35] Roberts DR , Chareonviriyaphap T, Harlan HH, et al 1997. Methods of testing and analyzing excito-repellency responses of malaria vectors to insecticides. J. Am. Mosq. Control Assoc. 13:13–17. 9152869

[tjag168-B36] Rydzanicz K , Jawień P, Lonc E, et al 2016. Assessment of productivity of *Culex* spp. larvae (Diptera: Culicidae) in urban storm water catch basin system in Wrocław (SW Poland). Parasitol. Res. 115:1711–1720. 10.1007/s00436-016-4912-x PMC4799245 26809340

[tjag168-B37] Sathantriphop S , Thanispong K, Sanguanpong U, et al 2014a. Comparative behavioral responses of pyrethroid–susceptible and–resistant *Aedes aegypti* (Diptera: Culicidae) populations to citronella and eucalyptus oils. J. Med. Entomol. 51:1182–1191. 10.1603/ME13191 26309305

[tjag168-B38] Sathantriphop S , White SA, Achee NL, et al 2014b. Behavioral responses of *Aedes aegypti*, *Aedes albopictus*, *Culex quinquefasciatus*, and *Anopheles minimus* against various synthetic and natural repellent compounds. J. Vector Ecol. 39:328–339. 10.1111/jvec.12108 25424262

[tjag168-B39] Senthil-Nathan S. 2020. A review of resistance mechanisms of synthetic insecticides and botanicals, phytochemicals, and essential oils as alternative larvicidal agents against mosquitoes. Front. Physiol. 10:1591. 10.3389/fphys.2019.01591 PMC7052130 32158396

[tjag168-B40] Strickman D , Lang JT. 1986. Activity of *Culex quinquefasciatus* in an underground storm drain in San Antonio, Texas. J. Am. Mosq. Control Assoc. 2:379–381. 3507518

[tjag168-B41] Suman LN , De S, Sharma G, et al 2022. Field larvicidal and oviposition repellent efficacy of three essential oil formulations for the control of filaria vector mosquito *Culex quinquefasciatus* Say (Diptera: Culicidae): a kill and push concept. J. Asia Pac. Entomol. 25:101963. 10.1016/j.aspen.2022.101963

[tjag168-B42] Sutthanont N , Sudsawang M, Phanpoowong T, et al 2022. Effectiveness of herbal essential oils as single and combined repellents against *Aedes aegypti*, *Anopheles dirus* and *Culex quinquefasciatus* (Diptera: Culicidae). Insects. 13:658. 10.3390/insects13070658 PMC9322308 35886836

[tjag168-B43] Walsh CJ , Roy AH, Feminella JW, et al 2005. The urban stream syndrome: current knowledge and the search for a cure. J. N. Am. Benthol. Soc. 24:706–723. 10.1899/04-028.1

[tjag168-B44] Wang X , Zhou G, Zhong D, et al 2021. Impact of underground storm drain systems on larval ecology of *Culex* and *Aedes* species in urban environments of Southern California. Sci. Rep. 11:12667. 10.1038/s41598-021-92190-3 PMC8209202 34135430

[tjag168-B45] Wetzel RG. 2001. Limnology: lake and river ecosystems. Gulf Professional. 10.1016/C2019-0-04412-3

[tjag168-B46] Withers PJA , Neal C, Jarvie HP, et al 2014. Agriculture and eutrophication: where do we go from here? Sustainability. 6:5853–5875. 10.3390/su6095853

[tjag168-B47] World Health Organization. 2013. Guidelines for efficacy testing of spatial repellents. WHO Press.

