# Supplementary material for: Multidimensional behavioral signatures of Culex quinquefasciatus (Diptera: Culicidae) in response to botanical volatiles reveal divergent patterns of mosquito displacement and behavioral disturbance
Source: J Med Entomol. 2026 Nov 6;63(6):tjag168. doi: 10.1093/jme/tjag168 (PMC13743431; doi:10.1093/jme/tjag168)
Supplement: Supplementary file 1 [file tjag168_supplementary_data.zip › Fig. S3 - Edited.pdf]

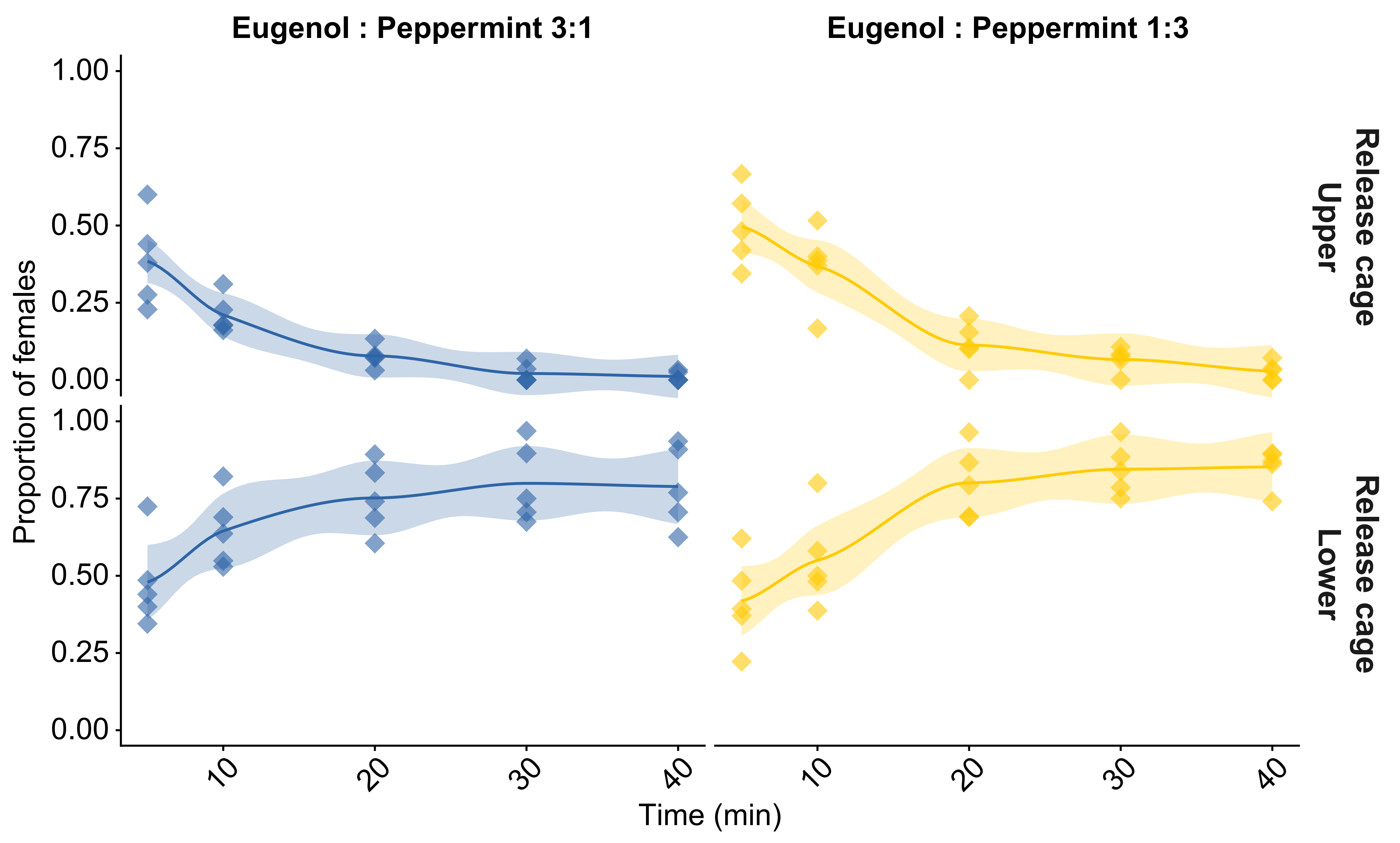
Eugenol : Peppermint 3:1
Eugenol : Peppermint 1:3
Release cage
Upper
Release cage
Lower
Proportion of females
0.00
0.25
0.50
0.75
1.00
10
20
30
40
Time (min)
