# Supplementary material for: Multidimensional behavioral signatures of Culex quinquefasciatus (Diptera: Culicidae) in response to botanical volatiles reveal divergent patterns of mosquito displacement and behavioral disturbance
Source: J Med Entomol. 2026 Nov 6;63(6):tjag168. doi: 10.1093/jme/tjag168 (PMC13743431; doi:10.1093/jme/tjag168)
Supplement: Supplementary file 1 [file tjag168_supplementary_data.zip › Supp. Info - Results_JME_Revision 1_JME_Clean Copy.docx]

**Supplementary Information - Tables:**

**Table S1. Commercial sources and product information for volatile treatments and controls used in the behavioral assays.**

| **Treatment/material** | **Material type** | **Supplier/manufacturer** | **Product or catalog number** | **Purity** |
| --- | --- | --- | --- | --- |
| Cinnamaldehyde | Individual plant-derived compound | Sigma-Aldrich, St. Louis, MO, USA  CAS Number: 104-55-2 | W228613 | ≥95% |
| Citronellol | Individual plant-derived compound | Sigma-Aldrich, St. Louis, MO, USA  CAS Number: 106-22-9 | W230901 | ≥95% |
| Eugenol | Individual plant-derived compound | Sigma-Aldrich, St. Louis, MO, USA  CAS Number: 97-53-0 | W246719 | ≥98% |
| Geraniol | Individual plant-derived compound | Sigma-Aldrich, St. Louis, MO, USA  CAS Number: 106-24-1 | W250716 | ≥97% |
| Geranium oil | Whole essential oil | Essential Oils of Egypt, Egypt | 100% Pelargonium graveolens | Undiluted essential oil |
| Peppermint oil | Whole essential oil | Sigma-Aldrich, St. Louis, MO, USA  CAS Number: 8006-90-4 | W284815 | Natural, Food grade |
| DEET | Synthetic positive control | W.C. Bradley Co., Columbus, GA, USA | Repel 100  Insect Repellent | 98.11%  DEET |
| Mineral oil | Negative control/carrier | Sigma-Aldrich, St. Louis, MO, USA  CAS Number: 8042-47-5 | M8410 | - |

Note: All materials were used as supplied by the manufacturer. Manufacturer-reported purity or compositional information is provided where available.

**Table S2. Pairwise comparisons of treatment-specific temporal slopes describing vertical redistribution of *Cx. quinquefasciatus* females within the release cage**

| **Treatment contrast** | **Difference in slope** | **SE** | **df** | ***t* ratio** | **Tukey-adjusted *P value*** |
| --- | --- | --- | --- | --- | --- |
| DEET − Cinnamaldehyde | 0.0036 | 0.0032 | 132.00 | 1.134 | 0.9164 |
| DEET − Citronellol | −0.0087 | 0.0032 | 132.00 | −2.727 | 0.0996 |
| DEET − Eugenol | −0.0107 | 0.0033 | 132.45 | −3.247 | **0.0243** |
| DEET − Geraniol | −0.0017 | 0.0034 | 132.00 | −0.497 | 0.9989 |
| DEET − Geranium oil | −0.0218 | 0.0032 | 132.00 | −6.793 | **<0.0001** |
| DEET − Peppermint oil | 0.0008 | 0.0031 | 132.00 | 0.274 | 1.0000 |
| Cinnamaldehyde − Citronellol | −0.0124 | 0.0032 | 132.00 | −3.862 | **0.0033** |
| Cinnamaldehyde − Eugenol | −0.0144 | 0.0033 | 132.45 | −4.348 | **0.0005** |
| Cinnamaldehyde − Geraniol | −0.0053 | 0.0034 | 132.00 | −1.566 | 0.7039 |
| Cinnamaldehyde − Geranium oil | −0.0254 | 0.0032 | 132.00 | −7.927 | **<0.0001** |
| Cinnamaldehyde − Peppermint oil | −0.0028 | 0.0031 | 132.00 | −0.911 | 0.9703 |
| Citronellol − Eugenol | −0.0020 | 0.0033 | 132.45 | −0.598 | 0.9968 |
| Citronellol − Geraniol | 0.0071 | 0.0034 | 132.00 | 2.074 | 0.3740 |
| Citronellol − Geranium oil | −0.0130 | 0.0032 | 132.00 | −4.065 | **0.0016** |
| Citronellol − Peppermint oil | 0.0096 | 0.0031 | 132.00 | 3.122 | **0.0350** |
| Eugenol − Geraniol | 0.0090 | 0.0035 | 132.41 | 2.587 | 0.1385 |
| Eugenol − Geranium oil | −0.0111 | 0.0033 | 132.45 | −3.349 | **0.0178** |
| Eugenol − Peppermint oil | 0.0116 | 0.0032 | 132.49 | 3.648 | **0.0068** |
| Geraniol − Geranium oil | −0.0201 | 0.0034 | 132.00 | −5.907 | **<0.0001** |
| Geraniol − Peppermint oil | 0.0025 | 0.0033 | 132.00 | 0.773 | 0.9871 |
| Geranium oil − Peppermint oil | 0.0226 | 0.0031 | 132.00 | 7.369 | **<0.0001** |

Temporal slopes in the table were estimated from a mixed-effects model of lower-versus-upper release-cage occupancy bias, with treatment, time, and their interaction included as fixed effects and replicate nested within assay date included as a random effect. The estimated difference is the slope for the first treatment minus that for the second treatment; positive values therefore indicate a steeper increase in lower-cage occupancy under the first treatment. Degrees of freedom were estimated using the Kenward–Roger method. *P* values were adjusted using Tukey's method for all pairwise comparisons among the seven treatment-specific slopes. Bold values indicate adjusted *P* < 0.05.

**Table S3. Mean 24-h mortality of Cx. quinquefasciatus females following volatile-treatment assays**

| **Treatment** | **Active tubes** | **Trials with mortality data / total trials** | **Mean mortality ± SE (%)** |
| --- | --- | --- | --- |
| Mineral oil (control) | 5 | 20/23 | 1.29 ± 0.55 |
| Cinnamaldehyde | 5 | 4/5 | 8.39 ± 1.98 |
| Citronellol | 5 | 4/5 | 4.29 ± 1.82 |
| DEET | 5 | 4/5 | 10.80 ± 6.46 |
| Eugenol | 1 | 4/5 | 0.89 ± 0.89 |
| Eugenol | 3 | 4/5 | 1.74 ± 1.02 |
| Eugenol | 5 | 0/5 | — |
| Geraniol | 5 | 0/4 | — |
| Geranium oil | 5 | 5/5 | 3.36 ± 1.83 |
| Peppermint oil | 1 | 5/5 | 0.65 ± 0.65 |
| Peppermint oil | 3 | 5/5 | 0.53 ± 0.53 |
| Peppermint oil | 5 | 6/6 | 15.15 ± 7.45 |
| Eugenol:Peppermint oil 3:1 | 3+1 | 5/5 | 5.10 ± 2.71 |
| Eugenol:Peppermint oil 1:3 | 1+3 | 5/5 | 0.00 ± 0.00 |
| Eugenol  (with sewer water volatiles) | 3 | 5/5 | 6.42 ± 3.39 |
| Eugenol:Peppermint oil 3:1 (with sewer water volatiles) | 3 + 1 | 5/5 | 34.91 ± 6.63 |

**Note:** Mortality was assessed 24 h after completion of each behavioral assay. For each independent assay trial, mortality was calculated as the number of dead females divided by the initial number of females in that trial. Values are the arithmetic mean ± standard error of the trial-level mortality percentages for trials with recorded 24-h mortality observations. Blank mortality entries were treated as missing data and were not assigned zero mortality. The numerator and denominator in “Trials with mortality data / total trials” indicate, respectively, the number of analyzed trials with recorded mortality outcomes and the total number of independent trials included in the behavioral analysis. Five-active-tube eugenol and peppermint-oil trials contributed to both the initial treatment screening and the active-tube exposure-level analysis but are presented only once. A dash indicates that no 24-h mortality observations were available for that treatment condition.

**Supplementary Information - Results:**

**Figure S1. Essential oils differentially structure vertical occupancy within the release cage.**

To resolve how essential oils influence the spatial organization of females that remained within the release cage, we examined the proportion of mosquitoes occupying the upper and lower cage sections over time (Fig. S1). Divergence between these trajectories reflects vertical redistribution within the release cage.

Vertical redistribution exhibited strong oil-specific temporal divergence. The significant oil-by-time interaction (*F*_6,132.17_ = 15.35, *P* = 2.70 × 10^-13^) indicates that oils generated distinct redistribution trajectories rather than a shared temporal pattern. Although overall vertical bias differed across oils (*F*_6,20.47_ = 6.30, *P* = 0.00071) and changed over the course of the assay (*F*_1,132.17_ = 61.34, *P* = 1.39 × 10^-12^), these effects were not independent. The direction and magnitude of temporal change varied substantially among oils, demonstrating that within-cage spatial organization is dynamically structured by oil identity.

Geranium oil produced the steepest positive temporal redistribution slope (*β* = 0.0231 ± 0.0023), reflecting progressive accumulation of females in the lower portion of the release cage over the 40 min assay. Eugenol (*β* = 0.0120 ± 0.0024) and citronellol (*β* = 0.0101 ± 0.0023) also exhibited significant positive slopes. In contrast, the estimated slopes for DEET (*β* = 0.0013 ± 0.0023), geraniol (*β* = 0.0030 ± 0.0025), peppermint oil (*β* = 0.0005 ± 0.0021), and cinnamaldehyde (*β* = −0.0023 ± 0.0023) were small and not statistically distinguishable from zero. Tukey-adjusted pairwise comparisons showed that the slope under geranium oil was significantly steeper than those under all other treatments (adjusted *P* ≤ 0.0178; Table S2). Eugenol also exhibited significantly steeper slopes than DEET (*P* = 0.0243), cinnamaldehyde (*P* = 0.0005), and peppermint oil (*P* = 0.0068), whereas citronellol exhibited significantly steeper slopes than cinnamaldehyde (*P* = 0.0033) and peppermint oil (*P* = 0.0350). No other pairwise comparisons were significant after Tukey adjustment.

These results show that volatile treatments differed not only in departure from the release cage but also in the vertical distribution of females that remained within it. Geranium oil, and to a lesser extent eugenol and citronellol, produced progressive shifts toward the lower portion of the release cage, whereas other treatments produced limited within-cage redistribution. Because this movement did not demonstrate directional displacement away from the volatile source, it is interpreted as within-chamber spatial reorganization rather than direct evidence of spatial repellency. Its ecological significance should be evaluated in larger semi-field or field environments.

**Figure S2. Treatment-specific vertical redistribution across active-tube exposure levels of eugenol and peppermint oil**

To determine whether active-tube exposure level influenced within-cage spatial organization, we quantified vertical redistribution under one-, three-, and five-active-tube conditions of eugenol and peppermint oil (Fig. S2). Mixed-effects analysis revealed significant main effects of time (*F*_1,115.19_ = 46.73, *P* = 4.11 × 10^-10^), active-tube exposure level (*F*_2,12.81_ = 5.45, *P* = 0.019), and treatment identity (*F*_1,12.81_ = 7.02, *P* = 0.020). A strong time × treatment interaction was also detected (*F*_1,115.19_ = 66.85, *P* = 4.31 × 10^-13^). In contrast, the time × active-tube exposure-level interaction (*P* = 0.593), treatment × active-tube exposure-level interaction (*P* = 0.597), and three-way interaction (*P* = 0.616) were not significant.

These results indicate that temporal vertical redistribution was structured primarily by treatment identity rather than by exposure-level-specific changes over time. Across all active-tube exposure levels, eugenol produced positive temporal slopes in lower-versus-upper cage occupancy bias (one tube: *β* = 0.0113 ± 0.0021; three tubes: *β* = 0.0142 ± 0.0021; five tubes: *β* = 0.0121 ± 0.0021), reflecting progressive accumulation of females in the lower portion of the release cage. In contrast, the slopes for peppermint oil were not distinguishable from zero at any active-tube exposure level, with all 95% confidence intervals overlapping zero, indicating limited temporal redistribution.

Although active-tube exposure level significantly influenced overall vertical occupancy bias, it did not alter the rate of redistribution over time. Thus, unlike the escape and flight-activity responses shown in Fig. 3, for which active-tube exposure level modified temporal trajectories, within-cage vertical reorganization remained broadly consistent across exposure levels within each treatment. Eugenol produced sustained downward redistribution under all three active-tube conditions, whereas peppermint oil produced little detectable temporal redistribution.

**Figure S3. Progressive vertical redistribution under eugenol–peppermint mixtures independent of blend ratio.**

Upon examining whether mixture composition altered within-cage vertical redistribution (Fig. S3), mixed-effects analysis revealed a strong effect of time (*F*_1,38_ = 106.78, *P* = 1.36 × 10^-12^), indicating progressive accumulation in lower cage strata across the assay period. In contrast, mixture identity did not significantly influence overall vertical bias (*F*_1,7.20_ = 3.33, *P* = 0.109), and the time × mixture interaction did not reach statistical significance (*F*_1,38_ = 3.39, *P* = 0.073).

Slope estimates confirmed positive temporal redistribution under both mixture formulations. The eugenol-dominant mixture (3:1) exhibited a positive redistribution slope (*β* = 0.0181 ± 0.0030), as did the peppermint-dominant mixture (β = 0.0259 ± 0.0030). Although the peppermint-dominant mixture (1:3) showed a numerically steeper slope, the difference between the two mixtures was not statistically significant (*P* = 0.073).

Thus, unlike flying behavior (Fig. 4B), where mixture composition produced strongly divergent temporal trajectories, vertical reorganization within the release cage followed broadly similar increases in lower-cage occupancy under both formulations. These results indicate that blending eugenol and peppermint did not generate qualitatively distinct vertical redistribution dynamics, and that within-cage spatial reorganization was driven primarily by time rather than mixture ratio.

**Figure S4. Time-dependent vertical redistribution persists under sewer-water volatile interference.**

Time-dependent vertical redistribution occurred under exposure to both eugenol-only and eugenol–peppermint (3:1) mixture in the presence of sewer-derived background volatiles (Fig. S4). Mixed-effects analysis revealed a strong effect of time (*F*_1,38_ = 67.14, *P* = 6.36 × 10^-10^), indicating progressive accumulation in lower cage strata across the assay period despite the presence of sewer volatiles. In contrast, odor source did not significantly influence overall vertical bias (*F*_1,16.38_ = 0.60, *P* = 0.451), and the time × odor interaction was not statistically significant (*F*_1,38_ = 3.25, *P* = 0.079).

Both treatments exhibited positive temporal redistribution slopes. Eugenol showed a significant increase in bottom bias over time (*β* = 0.0156 ± 0.00345), and the mixture exhibited a numerically steeper slope (*β* = 0.0244 ± 0.00345). However, the difference between slopes was not statistically significant (*P* = 0.079). These results indicate that although sewer-water volatiles attenuated escape and flying magnitudes (Fig. 6), they did not eliminate time-dependent vertical reorganization within the release cage, nor did they generate statistically distinct redistribution trajectories between odor sources.
